# Einsatz von KI-basierten Anwendungen durch Krankenhauspersonal: Aufgabenprofile und Qualifizierungsbedarfe

**DOI:** 10.1007/s00103-023-03817-x

**Published:** 2023-11-30

**Authors:** Dario Antweiler, Daniela Albiez, Dominik Bures, Bernadette Hosters, Florian Jovy-Klein, Kilian Nickel, Thomas Reibel, Johanna Schramm, Jil Sander, David Antons, Anke Diehl

**Affiliations:** 1 Fraunhofer Institut für Intelligente Analyse und Informationssysteme IAIS, Abteilung Knowledge Discovery, Schloss Birlinghoven 1, 53757 Sankt Augustin, Deutschland; 2 Fraunhofer Institut für Intelligente Analyse und Informationssysteme IAIS, Abteilung Adaptive Reflective Teams, Sankt Augustin, Deutschland; 3 Stabsstelle Digitale Transformation, Universitätsmedizin Essen, Essen, Deutschland; 4 Stabsstelle Entwicklung und Forschung Pflege, Universitätsmedizin Essen, Essen, Deutschland; 5 Institut für Technologie- und Innovationsmanagement, RWTH Aachen, Aachen, Deutschland

**Keywords:** Digitale Gesundheitskompetenzen, Künstliche Intelligenz, Qualifikation, Aufgabenprofil, Klinikpersonal, Digital Health Literacy, Artificial Intelligence, Qualification, Job profile, Clinical staff

## Abstract

**Hintergrund:**

Künstliche Intelligenz (KI) hat für Krankenhäuser wesentlich an Bedeutung gewonnen. Um die umfangreichen Potenziale der Technologie tatsächlich nutzen zu können, sind Anpassungen bei Aufgabenprofilen sowie zielgerichtete Qualifizierungsmaßnahmen für das Krankenhauspersonal heute und in Zukunft unabdingbar. Davon sind sowohl medizinische als auch nichtmedizinische Prozesse entlang der gesamten Wertschöpfungskette im Krankenhaus betroffen. Ziel der Arbeit ist es, einen Überblick über die notwendigen Fähigkeiten im Umgang mit intelligenten Technologien im klinischen Kontext zu geben und Maßnahmen zur Qualifizierung von Mitarbeiter*innen vorzustellen.

**Methoden:**

Im Rahmen des Projekts „SmartHospital.NRW“ wurden im Jahr 2022 eine Literaturrecherche sowie Interviews und Workshops mit Expert*innen durchgeführt. KI-Technologien und Anwendungsfelder wurden identifiziert.

**Ergebnisse:**

Zentrale Ergebnisse umfassen veränderte und neue Aufgabenprofile, identifizierte Synergien und Abhängigkeiten zwischen den einzelnen Aufgabenprofilen sowie die Notwendigkeit eines umfassenden interdisziplinären und interprofessionellen Austauschs beim Einsatz von KI-basierten Anwendungen im Krankenhaus.

**Diskussion:**

Unser Beitrag zeigt, dass Krankenhäuser frühzeitig Kompetenzen im Bereich Digital Health Literacy in der Belegschaft fördern und gleichzeitig technikaffines Personal anwerben müssen. Interprofessionelle Austauschformate sowie ein begleitendes Changemanagement sind essenziell für die Nutzung von KI im Krankenhaus.

**Zusatzmaterial online:**

Zusätzliche Informationen sind in der Online-Version dieses Artikels (10.1007/s00103-023-03817-x) enthalten.

## Abstract (en)

**Background:**

Artificial intelligence (AI) is becoming increasingly important for the future development of hospitals. To unlock the large potential of AI, job profiles of hospital staff members need to be further developed in the direction of AI and digitization skills through targeted qualification measures. This affects both medical and non-medical processes along the entire value chain in hospitals. The aim of this paper is to provide an overview of the skills required to deal with smart technologies in a clinical context and to present measures for training employees.

**Methods:**

As part of the “SmartHospital.NRW” project in 2022, we conducted a literature review as well as interviews and workshops with experts. AI technologies and fields of application were identified.

**Results:**

Key findings include adapted and new task profiles, synergies and dependencies between individual task profiles, and the need for a comprehensive interdisciplinary and interprofessional exchange when using AI-based applications in hospitals.

**Discussion:**

Our article shows that hospitals need to promote digital health literacy skills for hospital staff members at an early stage and at the same time recruit technology- and AI-savvy staff. Interprofessional exchange formats and accompanying change management are essential for the use of AI in hospitals.

## Hintergrund

Künstliche Intelligenz (KI) und die notwendige Digitalisierung treiben die Verbreitung einer datengetriebenen und personalisierten Behandlung von Patient*innen an. Krankenhäuser stehen im Zentrum des Prozesses, da sie einen Großteil der Behandlungsbereiche abdecken. Prominente Anwendungsfelder für KI im Krankenhaus umfassen Bilderkennung in der radiologischen Diagnostik, klinische Entscheidungsunterstützung, Arzneimitteltherapiesicherheit sowie Spracherkennung beim ärztlichen Diktat [1, 2]. Auch nichtmedizinische Prozesse können von einer KI-Unterstützung profitieren, darunter Informationsextraktion aus Dokumenten oder die Codierung im Medizincontrolling [3, 4]. Durch den Einsatz von KI-Anwendungen können Potenziale in den Bereichen Fehlervermeidung, Effizienz, Qualität sowie Zufriedenheit von Personal und Patient*innen erschlossen werden [4–6]. Zusätzlich kann die Digitalisierung zu einer gesteigerten positiven öffentlichen Wahrnehmung und Reputation der Krankenhäuser beitragen. Außerdem sind digitale und effiziente Krankenhäuser aufgrund der wachsenden Bedeutung von Nachhaltigkeit im Gesundheitswesen entscheidend, um messbare Fortschritte beim Umweltschutz zu erzielen [7, 8]. Anderseits kann es durch KI auch zu verstärkter Alarmmüdigkeit (Desensibilisierung durch viele, auch irrelevante Alarme) kommen [9] und komplexe Modelle erfordern eine Erklärbarkeit ihrer Ergebnisse [10].

Die steigende Integration von KI-Anwendungen führt zu einem Wandel des Arbeitsalltages im Krankenhaus. Dieser ist stark abhängig vom spezifischen Arbeitsbereich (Ambulanz, Station, OP-Saal etc.) sowie von der jeweiligen Datenverfügbarkeit und vorhandenen Interoperabilität. Aktuelle Literatur zeigt, dass die Nutzung von digitalen Technologien das Rollenverständnis von Mitarbeitenden stark verändert. Es ist zu beobachten, dass die Unsicherheiten durch den organisatorischen Wandel Stress und negatives Verhalten bei den Mitarbeiter*innen fördern [11–13]. Während diese negativen Auswirkungen breite Beachtung im wissenschaftlichen Diskurs finden, mangelt es an der Untersuchung potenzieller Lösungsansätze. Wir argumentieren, dass die Entwicklung relevanter Aufgabenprofile für die Nutzung von KI und die resultierenden Qualifizierungsbedarfe essenziell ist, um den Mitarbeitenden ein Verständnis über ihre neuen Rollen und Verantwortungen zu verschaffen. Deshalb untersuchen wir die folgenden Fragen:

- Welche Veränderungen ergeben sich für die Aufgabenprofile des Klinikpersonals durch den Einsatz von KI?
- Welche Qualifizierungen werden aufgrund der geänderten Aufgaben benötigt?
- Welche Maßnahmen können ergriffen werden, um diese Qualifizierungen umzusetzen?

Zur Beantwortung dieser Fragen haben wir Interviews und Workshops mit Expert*innen aus dem Gesundheitssektor im Rahmen des Projekts „SmartHospital.NRW“ durchgeführt. Die Ergebnisse unserer qualitativen Analyse umfassen 30 detaillierte Rollenprofile für den Umgang mit KI-Anwendungen im Krankenhaus. Unsere Arbeit erweitert den wissenschaftlichen Diskurs um einen Lösungsansatz für die anfallenden organisatorischen Änderungen infolge des Einsatzes von KI. Außerdem bieten unsere Ergebnisse praktische Implikationen, indem unsere Rollenprofile Krankenhausverantwortlichen die Vorbereitung der Mitarbeiter*innen auf anstehende Veränderungen im Tätigkeitsfeld ermöglichen.

### Forschungsstand.

Bisherige Arbeiten legen einen Fokus auf die Auswirkungen von KI auf die Aufgabenprofile einzelner Fachbereiche im Krankenhaus, darunter die Pflege [14–17], Psychiatrie [18], Onkologie [19] oder Radiologie [1, 20]. Deutlich wird, dass der Trend zum Einsatz von intelligenten und datenbasierten Systemen auch in Zukunft weiter zunehmen wird. Organisationen und Professionen im Gesundheitswesen werden sich dementsprechend grundlegend verändern [5, 17]. Mehrere Publikationen betonen die Notwendigkeit für mehr digitale Bildung in den Gesundheitsberufen und empfehlen die Integration in Ausbildung und Studium [14, 21]. Nach unserer Beobachtung fehlen in der Literatur eine strukturierte Analyse von KI-bezogenen Aufgabenprofilen anhand klinischer Bedarfe, eine Übersicht von Klinikprozessen mit Einsatzmöglichkeiten für KI sowie eine Abbildung von KI-Technologiebedarfen auf Tätigkeiten und Fähigkeiten des Klinikpersonals.

## Methoden

**Figure 1:**
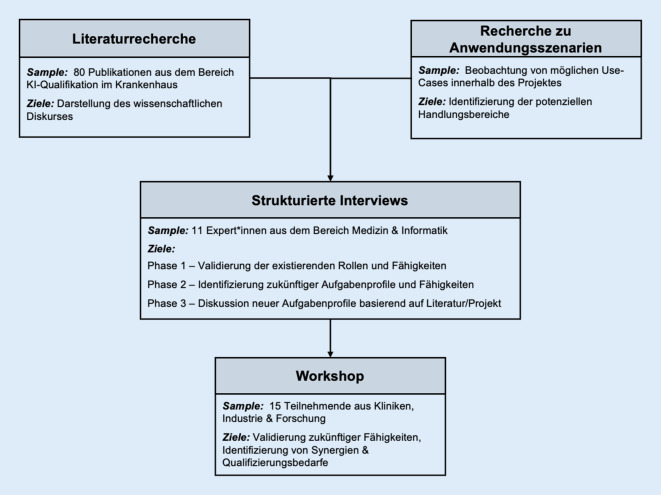

Eine Übersicht zu den in dieser Studie eingesetzten Methoden, zu deren Abfolge und zu den jeweiligen Zielen bietet Abb. 1.

### Literaturrecherche.

Zu Beginn wurden mittels einer Literaturrecherche inhaltlich verwandte Publikationen identifiziert und ausgewertet. Dazu gehören fachbezogene Monografien zu den Themen „Smart Hospital“ und „Future Skills in Medizin und Gesundheit“ [4, 11], inkl. darin referenzierter Primärliteratur, sowie wissenschaftliche Fachpublikationen, die durch geeignete Suchbegriffe, wie z. B. „hospital ai qualifications“, auf den Publikationsplattformen PubMed und Google Scholar gefunden wurden. Außerdem wurden Studien, Whitepaper und Projektberichte, die sich mit der Kombination der 3 Themengebiete „Krankenhaus“, „Digitalisierung“ und „Qualifikation“ befassen, betrachtet [22, 23]. Insgesamt wurden 80 Publikationen in die Analyse miteinbezogen, die bis Februar 2023 publiziert wurden (Onlinematerial 1).

### Strukturierte Interviews.

Es wurden Interviews mit 11 Expert*innen von 2 Einrichtungen aus den Bereichen Pflege, Ärzteschaft, Medizincontrolling, Personalplanung, OP-Bereich, IT und Leitung anhand strukturierter Leitfäden durchgeführt (vgl. Onlinematerial 2, 3). Diese wurden protokolliert, anonymisiert und deduktiv entlang von Leitfragen ausgewertet. Sie dienen als Grundlage für die Identifikation von Aufgabenprofilen und Qualifizierungsbedarfen. Jedes Interview bestand dabei aus 3 Phasen: Nach einer Vorstellung wurde in der 1. Phase der bisherige Einsatz von digitalen Technologien im Tätigkeitsfeld der Person thematisiert. Der Fokus lag auf bereits existierenden Rollen sowie deren Fähigkeiten und Einsatz entlang des Behandlungsverlaufs. Die 2. Phase beschäftigte sich mit dem Ausblick auf die Zukunft. Dort wurde abgefragt, welche neuen Technologien wahrscheinlich in die klinische Praxis einziehen und welche Rollen davon betroffen sein werden. Teilweise wurden neue Aufgabenprofile genannt, die notwendig sein werden. In der abschließenden 3. Phase wurden neue und veränderte Aufgabenprofile diskutiert, welche in der Literatur, vorangegangenen Projekten oder Interviews identifiziert wurden. Insgesamt wurden 30 Aufgabenprofile entwickelt. Dafür wurden identifizierte Fähigkeiten und Tätigkeiten analysiert, harmonisiert und zu Profilen zusammengeführt.

### Expert*innen-Workshop.

Im Rahmen des Forschungsprojektes wurde im Jahr 2022 eine Fachkonferenz mit Expert*innen aus der Gesundheitsbranche durchgeführt. Vertreten waren Personen aus Kliniken, Wissenschaft, Industrie sowie Start-ups. Zum Thema „Aufgabenprofile und Qualifizierungsbedarfe im Krankenhaus der Zukunft“ wurde ein Workshop mit 15 Teilnehmenden durchgeführt. In der Gruppe waren u. a. ärztliches, pflegerisches und leitendes Personal aus Krankenhäusern sowie Wissenschaftler*innen aus Informatik und Wirtschaft vertreten. Die Teilnehmenden erarbeiteten in Kleingruppen zukünftig relevante Fähigkeiten, Prozesse, Synergien, Technologien und Qualifizierungsbedarfe zu je einer Berufsgruppe.

### Anwendungsszenarien und Potenzialanalyse.

**Figure 2:**
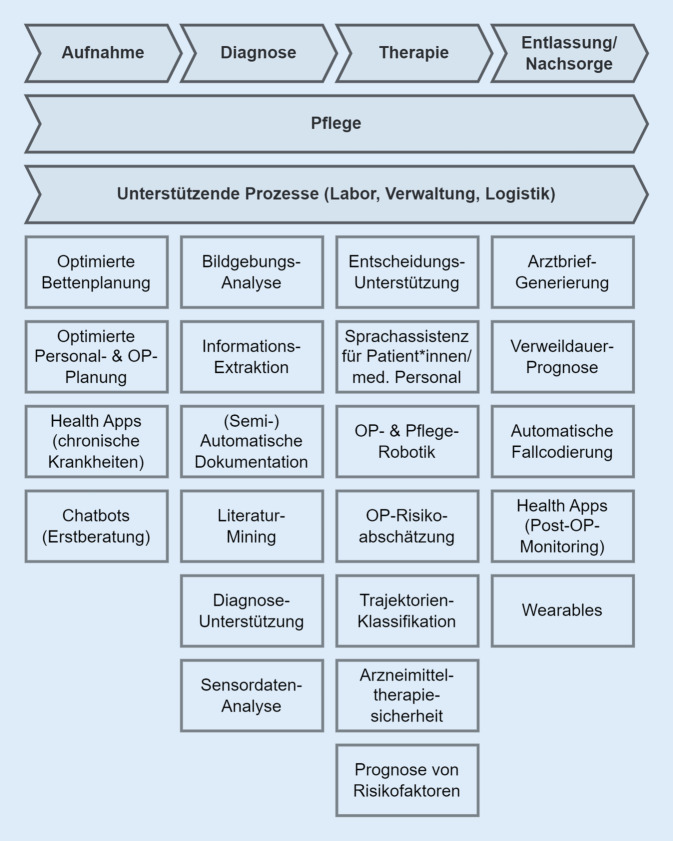

Eine weitere Quelle für Qualifizierungsbedarfe stellen Anwendungsszenarien (*Use Cases*) dar. Dabei handelt es sich um Potenzialfelder, bei denen eine bestimmte KI-Technologie oder Kombination von Technologien^1^ eine klar umrissene Herausforderung adressiert und eine messbare Unterstützung bietet. Zunächst wurden projektinterne Use Cases als Ausgangspunkt verwendet. Durch umfangreiche Recherchen wurden weitere Szenarien identifiziert, bis keine neuen mehr hinzugekommen sind. Anschließend wurden diese Szenarien aufgeführt, analysiert, zusammengefasst und auf vorhandene Aufgabenprofile abgebildet. Dabei erfolgte die Gruppierung auf Basis von ähnlichen Technologien, gemeinsamen Berufsgruppen oder prozessualer Nähe (Abb. 2).

## Ergebnisse

### Literaturrecherche

**Figure 3:**
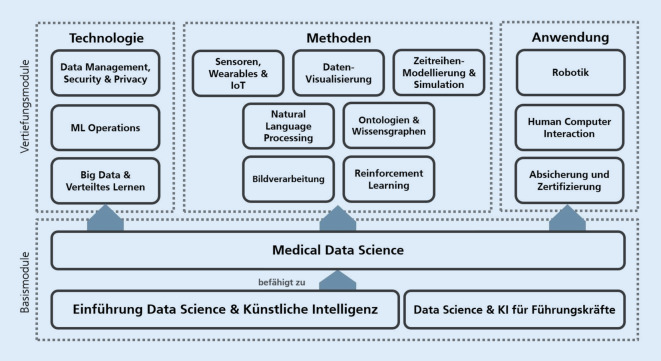

Aus dem wissenschaftlichen Diskurs wird deutlich, dass spezifische Qualifizierungsbedarfe für das Personal definiert werden sollten, um die genannten Aufgabenprofile mit den entsprechenden Fähigkeiten und Kenntnissen abzubilden. Zum Erwerb der notwendigen Qualifizierungen eignen sich Lernformen, die sich je nach Schulungsbedarf oder persönlichen Präferenzen gestalten oder kombinieren lassen. Für die weitere Qualifizierung sind Fachvorträge, Planspiele, Lernplattformen, Data Labs, Blended Learning, Feedbackrunden, Hackathons, Peer Teachings und Massive Open Online Courses (MOOCs) nutzbar [21, 24–26]. Ein beispielhaftes Schulungskonzept wurde im Projekt entwickelt und ist in Abb. 3 dargestellt. Die Nachfrage nach digitaler Bildung ist unter ärztlichem Personal und Medizinstudent*innen hoch. Die Nutzungsintensität der Angebote sollte regelmäßig durch Assessments gemessen werden [27].

### Anwendungsszenarien

Als Teilergebnis des laufenden Projektes wurden 38 Use Cases für den KI-Technologieeinsatz identifiziert (Onlinematerial 4, 5). Diese wurden mit projektinternen Expert*innen geclustert und in Abb. 2 visualisiert. Die Darstellung entlang einer Wertschöpfungskette wurde aus Gründen der Vereinheitlichung gewählt. Die Hauptwertschöpfung des Krankenhauses besteht in der Versorgung der Patient*innen. Die Pflege und unterstützende Prozesse wie Labor, Verwaltung und Logistik sind querschnittliche Tätigkeiten. Darüber hinaus ist die Wertschöpfungskette in Aufnahme, Diagnose, Therapie und Entlassung/Nachsorge aufgeteilt. Zugleich gibt es weitere sekundäre und unterstützende Tätigkeiten sowie vor- und nachgelagerte Prozessschritte.

Die KI kann die Betten‑, Personal- und OP-Planung unterstützen. Über intelligente Chatbots kann die Aufnahme vereinfacht werden, indem Vorerkrankungen und Symptome abgefragt werden. KI kann bei der Auswertung von Daten aus Health Apps helfen. In der Radiologie können bildgebende Verfahren mit KI unterstützt werden [1, 3]. Weiterhin können Informationen aus Dokumenten extrahiert, Dokumentationen ergänzt oder relevante Literatur gefunden werden. In der Therapie können Entscheidungen unterstützt werden, wie im Projekt *Sepsis Watch* exemplarisch zu sehen ist [2]. Mit Sprachassistenten besteht die Möglichkeit, Geräte zu steuern oder die notwendige Dokumentation während der Therapie zu automatisieren; im OP und bei der Pflege kann KI-basierte Robotik unterstützen. Ebenso kann bei Verabreichung von Arzneimitteln eine höhere Sicherheit gewährleistet werden [3]. Weiterhin ergeben sich auch Anwendungsmöglichkeiten für das Entlassmanagement und die Nachsorge. Dazu zählt die Verweildauerprognose, die Generierung von Arztbriefen, die automatische Fallcodierung sowie eine Auslastungsoptimierung bei Überweisungen [28].

### Strukturierte Interviews

**Figure 4:**
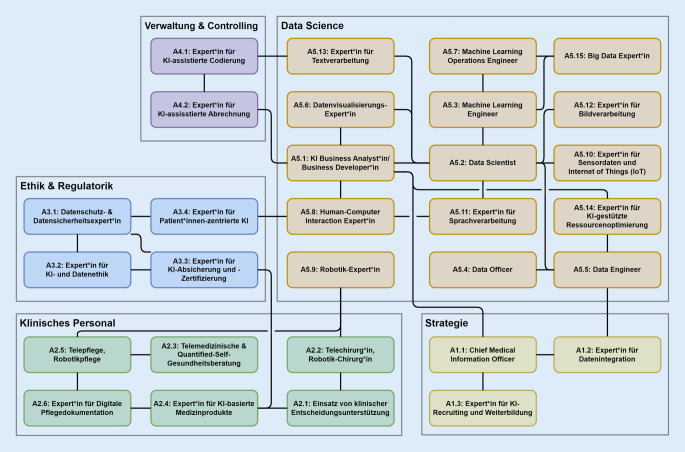

Die in der 2. Phase der Interviews hergeleiteten Aufgabenprofile lassen sich auf Basis der anschließenden Diskussion mit den Interviewten (3. Phase) in 5 Bereiche unterteilen. Jedes Aufgabenprofil referenziert existierende Aufgabenprofile sowie die damit assoziierten Fähigkeiten und beschreibt die veränderten bzw. zusätzlich benötigten Fähigkeiten (abgeleitet aus der 1. Phase der Interviews; vgl. Onlinematerial 6). Die Aufgabenprofile sind zusammen mit ihren jeweiligen Synergien und Abhängigkeiten zu anderen Profilen in Abb. 4 visualisiert. Im Folgenden werden die Aufgabenprofile vorgestellt.

#### Strategie.

Aus den Interviews geht hervor, dass die Anwendung von KI nahezu alle Personengruppen betreffen wird und disziplinübergreifende Interaktion benötigt. Daher sind sowohl eine KI-Strategie als auch eine koordinierende Stelle im Krankenhaus für eine erfolgreiche digitale Transformation unabdingbar. Solch eine Rolle wird z. B. als Chief Transformation Officer (CTO)^2^ definiert und ist in der Regel mit dem Vorantreiben von digitalen Innovationen im Krankenhaus verknüpft. Eng damit verbunden sind die Rollen für Recruiting und Weiterbildung, um digitalaffinen Nachwuchs anzuwerben, zu fördern und zu halten sowie Verantwortliche für das Thema Datenintegration zu identifizieren.

#### Data Science.

Die Aufgabenprofile im Data-Science-Cluster lassen sich in 2 Gruppen teilen, eine entlang der Phasen des CRISP-DM^3^ [30] und eine bestehend aus technologiespezifischen Profilen. Den Phasen entsprechen die Rollen Business Developer, Data Officer & Data Engineer, Data Scientist, Machine-Learning Engineer, Machine-Learning-Operations Engineer und Datenvisualisierung. Sie arbeiten als Team in einem Data-Science-Projekt zusammen, um einen Use Case von einer Geschäftsidee zu einer laufenden KI-Anwendung zu entwickeln. Die spezifischeren Profile sind bei bestimmten Datentypen und technischen Herausforderungen relevant. Dazu zählen die Themen Sprache, Texte, Bilder, Sensordaten, Robotik, Big Data [31] sowie Mensch-Maschine-Interaktion, Usability und Technologieakzeptanz [32, 33].

#### Klinisches Personal.

Sowohl Ärzt*innen als auch Pfleger*innen werden in Zukunft vermehrt Entscheidungsunterstützungssysteme (Clinical Decision Support Systems – CDSS) einsetzen. Damit werden digitale Tools bezeichnet, die auf Basis der vorhandenen Daten eine Empfehlung für Diagnose oder Therapie aussprechen. Aus den Interviews und der Literaturrecherche ergab sich die Anforderung, dass medizinisches Personal die Ergebnisse der KI sowohl mit eigenem Wissen und Erfahrung als auch mit Behandlungsleitlinien abgleichen muss. Darauf basierend muss entschieden werden, ob die Empfehlung des Systems umgesetzt wird. Die Mitarbeitenden werden sich vermehrt mit Datenquellen wie Wearables und Telemedizin für die interne wie externe Kommunikation auseinandersetzen [9]. Besonders die Chirurgie und Pflege wird von Robotik-Anwendungen profitieren [15]. Automatische Dokumentation und digitale Assessmenttools unterstützen Pflegefachpersonen bei der Ermittlung des individuellen Pflegebedarfs und der Auswahl evidenzbasierter Pflegeinterventionen. Mit zunehmendem KI-Einsatz verändern sich die klinischen Rollen, die sich vermehrt durch die Vermittlung zwischen Patient*innen und digitalen Systemen definieren [34]. Um den neuen Anforderungen kompetent begegnen zu können, sind Qualifizierungen in den Bereichen Telemedizin, Robotik und KI-basierte Medizinprodukte notwendig. So können diese Technologien partizipativ mit den Pflegebedürftigen in deren Behandlungsprozess eingebunden werden [14, 16, 17].

#### Verwaltung und Controlling.

Unterstützende Prozesse im Krankenhaus können von KI-Methoden profitieren. Besonders der Logistikbereich erfährt durch Ressourcenplanung, Lieferkettenanalyse, intelligente Inventarisierung und vorausschauende Instandhaltung ein erhebliches Einsparpotenzial [35]. Dadurch werden positive Effekte in der gesamten Infrastruktur eines Krankenhauses sichtbar. Außerdem hat KI einen starken Einfluss auf das Medizincontrolling. Durch Kostendruck und Fallpauschalen stehen Controller finanziell im Fokus eines jeden Hauses und produzieren mithilfe von KI-Unterstützung Abrechnungen, die qualitätssicher und kostenoptimiert sind.

#### Ethik und Regulatorik.

Von den Interviewten wird außerdem hervorgehoben, dass die Datenbasis für das Kollektiv, auf das KI angewendet wird, repräsentativ sein muss und keine statistischen Verzerrungen (Bias) enthalten darf. Die Detektion und Mitigation von Bias sowie hohe Anforderungen an Informationssicherheit sollten im Fokus dedizierter KI-Aufgabenprofile liegen. Dazu zählen Expert*innen für Absicherung und Zertifizierung von KI, für KI- und Datenethik sowie die bereits existierenden Datenschutz- und Informationssicherheitsverantwortlichen. Ein besonderes Augenmerk liegt auf Anwendungen, die als Medizinprodukt unter die Anforderungen der „Medical Device Regulation“ fallen und zukünftig ggf. durch das „Gesetz über künstliche Intelligenz“ der Europäischen Union (EU AI Act) reguliert werden [36].

### Workshop

Ein zentrales Ergebnis der strukturierten Gruppendiskussionen im Workshop ist die Validierung der identifizierten (nicht-)technischen Fähigkeiten, die das Krankenhauspersonal zukünftig beherrschen muss. Diese beziehen sich sowohl auf den professionellen Umgang mit Sensoren, Apps und Sprachtechnologien als auch auf Empathie, Kommunikation und Interdisziplinarität. Auf Basis der Workshopergebnisse konnten Synergien zwischen den Bereichen, insbesondere durch Querschnittsthemen wie Telemedizin, Robotik und Medizinprodukte, von denen mehrere Organisationseinheiten eines Krankenhauses profitieren, identifiziert werden (Abb. 4). Eine erfolgreiche Umsetzung von Data-Science-Projekten nach dem CRISP-DM-Zyklus basiert auf einer intensiven Zusammenarbeit zwischen Domänenexpert*innen auf der einen Seite, die eine fachliche Perspektive mitbringen und dabei Daten und Ergebnisse interpretieren sowie Evaluation durchführen können. Auf der anderen Seite stehen Data Scientists mit technischer Expertise und Modellierungserfahrung. Um KI effektiv nutzen zu können, bedarf es einer zugrunde liegenden IT-Strategie. Diese sollte insbesondere eine enge Zusammenarbeit zwischen Entwickler*innen und klinischen Verantwortlichen sowie die kontinuierliche Evaluation der Anwendungen umfassen [37]. Es ist essenziell, die Identität, Werte und Arbeitsweisen des medizinischen Personals zu berücksichtigen, da diese maßgeblich für Herausforderungen bei der Implementierung von IT-Anwendungen sind [38]. Dies gilt insbesondere im Kontext von KI-Anwendungen, da KI ein spezifisches Risiko für Identitätsverluste bei Mitarbeitenden darstellt [39]. Themen wie Informationssicherheit, Ethik in KI und Projektmanagement müssen fächerübergreifend betrachtet werden [15]. Zudem sind eine positive Fehlerkultur, die Vermittlung zwischen Maschine und Mensch und rechtliches Wissen essenziell.

### Triangulation

Es wurde explizit eine Kombination von Methoden angewendet, um das Thema auf Basis unterschiedlicher Daten- und Methodenquellen zu analysieren. Dadurch konnten übergreifende Themen identifiziert und diese aus verschiedenen Perspektiven validiert werden. Folgende notwendige Fähigkeiten für das Krankenhauspersonal konnten aus der Analyse der Interviews, dem Workshop im Rahmen der Fachkonferenz sowie den Use Cases gewonnen werden:

- **Datenkompetenz**: Es besteht ein hoher Bedarf für Sachverständnis im Umgang mit Daten.
- **Digitale Gesundheitskompetenz (Digital Health Literacy)**: Digitale Technologien entwickeln sich dynamisch, der souveräne Umgang muss stetig trainiert werden.
- **Digitale Omnipräsenz**: Digitalisierung betrifft alle organisatorischen Einheiten im Krankenhaus entlang der Wertschöpfungskette.
- **Interdisziplinarität und -professionalität**: Übergreifende Zusammenarbeit der Berufsgruppen und Fachbereiche ist von immenser Bedeutung.
- **Qualifizierungen**: Digitale Gesundheitskompetenz muss früh aufgebaut und praxisnah in die Ausbildung von Gesundheitsfachberufen integriert werden.
- **Meta-Skills**: Expertise zu Ethik, Recht, Informationssicherheit, Patient*innen-Kommunikation [5], Verarbeitung komplexer Informationen und kritische Analyse von KI-Vorschlägen.

## Diskussion

### Studium und Ausbildung zum Erwerb digitaler Kompetenzen

In den aktuellen Curricula der gesundheitsbezogenen Studiengänge und Ausbildungen in Deutschland sind digitale Kompetenzen nicht ausreichend abgebildet [5, 7, 14, 15]. Auch nach Reformierung der Ausbildungsordnung und des Pflegeberufegesetzes findet die Vermittlung von digitalen Kompetenzen in der pflegerischen Ausbildung weiterhin kaum Berücksichtigung. Digitale (Gesundheits‑)Kompetenzen müssen jedoch in der Gruppe der Pflegeberufe vorhanden sein, damit zukünftig Systeme nicht nur verwendet, sondern auch verstanden werden. Zusätzlich nehmen professionell Pflegende eine wichtige Schlüsselfunktion im Rahmen des *Shared Decision Making* ein, indem sie digitale Kompetenzen an Patient*innen und Angehörige vermitteln und Hintergründe erläutern [40]. Auch die Vermittlung dieser Fähigkeit wird essenziell werden.

Deshalb wird die Schaffung von neuen Studiengängen wie „Pflege und Digitalisierung“ [41] oder eine curriculare Integration entsprechender Module in Studiengänge und deren Integration in nationale Kompetenzkataloge [42] wichtig. Die Einbettung moderner Lernangebote in die Bildungskonzepte sollte daher bereits während der Ausbildung und im Studium niederschwellig erfolgen und homogen in die Krankenhauslandschaft für die berufliche Weiterqualifizierung eingegliedert werden. Eine Gesamtstrategie fehlt indes. Nationale und internationale Kommissionen empfehlen eine stärkere Eingliederung dieser Themen und betonen digitale Fähigkeiten als Kernkompetenz [41, 43–46]. Ergänzend zu der Integration der Thematik in Ausbildung und Studium gilt es, Pflegefachpersonen weiterzubilden.

### Auswirkungen von KI-Technologien auf die Praxis

Mit vermehrtem Einsatz von KI-Technologien geht eine Zunahme an Aufgabenkomplexität vieler Rollen und Fachberufe einher. Durch den Einsatz von KI etablieren sich neue Teamstrukturen, Hierarchien und Prozesse. Die Teilautomatisierung durch KI birgt das Risiko für Angst vor Arbeitsplatzverlust, fördert jedoch auch die Entstehung von neuen Tätigkeitsprofilen. Die KI-bedingten Auswirkungen auf den Arbeitsmarkt sind stark von der Art der unterstützten Tätigkeit und Anpassungsfähigkeit der Arbeitnehmer*innen abhängig. So beeinflusst die Nachfrage nach bestimmten Arbeitskräften und -bereichen die Auswirkungen von KI auf den Arbeitsmarkt [47]. In gegenwärtigen Digitalstrategien der Krankenhäuser steht vor allem die Prozessoptimierung im Fokus. Das Ziel besteht darin, medizinische Abläufe digital zu erfassen und Daten bereitzustellen, um eine sichere und effiziente Behandlung zu gewährleisten [48].

Am Beispiel der oben beschriebenen erwarteten Entwicklungs- und Einsatzvermehrung von CDSS in klinischen Bereichen ergibt sich ein Qualifizierungsbedarf für Krankenhäuser nicht erst zum Einsatzzeitpunkt, sondern deutlich vorher. Je mehr eine KI-Anwendung auf Daten unterschiedlicher Quellen operiert, desto mehr müssen Mitarbeitende aus verschiedenen Fachbereichen interdisziplinär zusammenarbeiten. Ein konkretes Beispiel hierfür ist eine KI-gestützte OP-Planung, bei der viele Randbedingungen und Akteur*innen simultan berücksichtigt werden müssen.

### Risiken von KI müssen berücksichtigt werden

Die Ergebnisse dieser Arbeit zeigen, dass KI neben dem großen Potenzial für Wissenschaft und Gesundheit Risiken birgt. Die befragten Expert*innen betonen die Wichtigkeit, systematische Verzerrungen in Daten, Algorithmen und ihrer Anwendung zu minimieren. Auch die Weltgesundheitsorganisation (WHO) hebt die Bedeutung von Gerechtigkeit und Vorurteilsfreiheit in der KI hervor [49]. Darüber hinaus müssen bei der Entwicklung großer Sprachmodelle negative Aspekte beachtet werden, wie z. B. die Möglichkeit, dass die Modelle Textgenerierungen produzieren, die halluziniert sind, also beispielsweise nicht mit klinischen Werten übereinstimmen. Daher ist es wichtig, sorgfältige Evaluierungsrahmen zu entwickeln, um Fortschritte zu messen, potenzielle Schäden zu erfassen und zu mildern [50].

### Limitationen und Implikationen

Die Ergebnisse dieser Studie wurden primär im Rahmen des Forschungsprojektes SmartHospital.NRW erarbeitet. Die Auswahl von Interview- und Workshop-Teilnehmer*innen kann dazu führen, dass Qualifizierungs- und Schulungsbedarfe für die Übertragung auf andere Krankenhäuser angepasst werden müssen. So ist die Universitätsmedizin Essen als Maximalversorger mit angeschlossenem KI-Institut hinsichtlich KI-Nutzung vergleichsweise gut aufgestellt. Krankenhäuser, die am Anfang der digitalen Transformation stehen, können andere Gewichtungen und Priorisierungen aufweisen. Außerdem können sich die Aufgabenprofile und Qualifizierungsbedarfe, die in dieser Studie ermittelt wurden, aufgrund des digitalen und technologischen Fortschritts ändern. Es wird empfohlen, die Ergebnisse regelmäßig zu bewerten und an die technologische Entwicklung anzupassen, darunter beispielsweise große Sprachmodelle^4^.

Des Weiteren ist die Reihenfolge der Datenerhebung kritisch zu reflektieren. Wenn strukturierte Interviews zuerst durchgeführt werden und ihre Ergebnisse anschließend in Workshops zur Validierung vorgestellt werden, entsteht ein Risiko für eine Bestätigungsverzerrung. Die Workshop-Teilnehmer*innen könnten dazu neigen, den bereits erarbeiteten Aufgabenprofilen zuzustimmen oder ihre Meinungen daran anzupassen, insbesondere wenn diese Profile als Basis für die Diskussion verwendet werden. Um dieses Problem zu adressieren, wurden in den Workshops eine offene Diskussion gefördert und die Teilnehmer*innen ermutigt, sowohl bestätigende als auch abweichende Ansichten zu äußern. Auf Basis der dargelegten Aufgabenprofile ergibt sich ein Bedarf an zukünftigen Forschungsvorhaben, insbesondere in Hinblick auf die Untersuchung von Synergieeffekten zwischen Mitarbeiter*innen.

Die Gesamtheit der vorgestellten Aufgabenprofile beschreibt einen Idealzustand, der unter Berücksichtigung der jeweiligen lokalen Umstände zu erfüllen ist. Krankenhäuser müssen eine individuelle Priorisierung ihres Qualifizierungsbedarfs vornehmen und können mehrere Aufgabenprofile durch eine Person oder durch externe Zusammenarbeit abdecken. Weitere Untersuchungen sollten klären, ob Aufgabenprofile für unterschiedliche Krankenhaustypen vereinfacht, kombiniert oder erweitert werden sollten.

Schließlich wurde eine Übersicht für Qualifizierungsbedarfe dargelegt. Dieser Ansatz soll dazu anregen, sich systematisch und Use-Case-getrieben mit Entwicklungsbedarfen zu beschäftigen. Schulungskonzepte sollten entsprechend einer Strategie für den zielgerichteten Einsatz von KI entwickelt werden. Es ergeben sich folgende Implikationen für anknüpfende Forschung:

- **Schulungskonzepte**: Entwürfe von Schulungskonzepten sind weiter auszuarbeiten, in einem praktischen Rahmen zu erproben und entsprechend anzupassen.
- **Wirkungsanalyse:** Strategischer KI-Einsatz verändert Rollen und Aufgaben. Zukünftige Studien sollten erforschen, welche Veränderungen und Auswirkungen sich für Mitarbeiter*innen und Patient*innen ergeben.

## Fazit

Durch die Studienergebnisse ergibt sich ein klares Bild für die Anforderungen an die Qualifikationen der Mitarbeitenden für den Einsatz von KI im Krankenhaus:

- Neue und veränderte Aufgabenprofile entstehen entlang der gesamten Wertschöpfungskette bei (nicht‑)​medizinischen Prozessen.
- Eine Vielfalt von Kompetenzen und Fähigkeiten ist notwendig (technisch, sozial, organisatorisch) und kann nur über gemischte Teams erreicht werden.
- Die erfolgreiche Nutzung von KI-Anwendungen benötigt Veränderungen in der Organisationsstruktur und -kultur; Veränderungsmanagement (Changemanagement) soll die Mitarbeitenden einbeziehen.

Aus den erfassten Beobachtungen ergeben sich konkrete Handlungsempfehlungen für Entscheidungsträger*innen in Krankenhäusern:

- Die Transformation zu einem „Smart Hospital“ muss rechtzeitig durch die Entwicklung und Kommunikation einer umfassenden (Digitalisierungs‑)Strategie angestoßen werden
- Mitarbeiter*innen aus allen Berufsgruppen und Fachbereichen sind frühzeitig in die Entwicklung von KI-Anwendungen einzubinden.
- Netzwerke mit anderen Kliniken, Unternehmen und Stakeholdern müssen gebildet werden.
- Im Krankenhaus muss ein Bewusstsein für digitale Transformation, KI und Interoperabilität geschaffen werden, welches den Aufbau von Bildungsangeboten integriert.

Zentrale Herausforderungen bei der Umsetzung umfassen die enorme Geschwindigkeit der technischen Entwicklung, mit der die Qualifizierung der Mitarbeitenden mithalten muss, die enorme Breite des Themas KI im Krankenhaus sowie den Aufbau multiprofessioneller Austauschformate für die Entwicklung und den Einsatz von KI vor dem Hintergrund des Fachkräftemangels.

### Supplementary Information





## Footnotes

1 Mögliche Technologien: Entscheidungsunterstützungssysteme, Patientenportale, elektronische Akten, Telemedizin, Mobile Apps, Augmented und Virtual Reality, Robotik, Wearables und Sensorik. Methoden spezifisch für die KI sind u. a. Bild‑, Sprach- und Textverarbeitung sowie Datenvisualisierung.

2 Verwandte Rollenbezeichnungen lauten u. a. „Chief (Medical) Information Officer“ (CMIO). CMIO als Begriff stammt aus dem britischen National Health Service (nationaler Gesundheitsdienst) und ist ein Ausbildungsgang für Mediziner*innen, der Kompetenzen im Bereich Digitalisierung/Informatik umfasst. CIOs sind im Regelfall (Medizin‑)Informatiker*innen. Die Position ist meist mit vertragsrechtlich verknüpften Haftungsklauseln für IT-Ausfälle verknüpft [29].

3 Industrieweites Standardmodell für Datenanalyseprozesse.

4 https://openai.com/blog/chatgpt/; Stand 15.08.2023.

## References

[1] Schaffter T, Buist DSM, Lee CI (2020). Evaluation of combined artificial intelligence and radiologist assessment to interpret screening mammograms. JAMA Netw Open.

[2] Sendak MP, Ratliff W, Sarro D (2020). Real-world integration of a sepsis deep learning technology into routine clinical care: implementation study. JMIR Med Inform.

[3] Broich K, Löbker W, Lauer W (2021). Beitrag des BfArM zur Potenzialentfaltung der Digitalisierung im Gesundheitswesen – digital readiness@BfArM. Bundesgesundheitsbl.

[4] Werner JA, Forsting M, Kaatze T, Schmidt-Rumposch A (2020). Smart hospital.

[5] Schneider D, Sonar A, Weber K, Pfannstiel MA (2022). Zwischen Automatisierung und ethischem Anspruch – Disruptive Effekte des KI-Einsatzes in und auf Professionen der Gesundheitsversorgung. Künstliche Intelligenz im Gesundheitswesen.

[6] Stead S, Vogt L, Antons D (2023). Hospital resource endowments and nosocomial infections: longitudinal evidence from the English national health system on clostridioides difficile between 2011 and 2019. J Hosp Infect.

[7] Werner JA, Kaatze T, Schmidt-Rumposch A (2022). Green Hospital: Nachhaltigkeit und Ressourcenschonung im Krankenhaus.

[8] Salge TO, Antons D, Barrett M (2022). How IT investments help hospitals gain and sustain reputation in the media: the role of signaling and framing. Inf Syst Res.

[9] Lambert SI, Madi M, Sopka S (2023). An integrative review on the acceptance of artificial intelligence among healthcare professionals in hospitals. Npj Digit Med.

[10] Di Martino F, Delmastro F (2023). Explainable AI for clinical and remote health applications: a survey on tabular and time series data. Artif Intell Rev.

[11] Matusiewicz D, Werner JA (2021). Future Skills in Medizin und Gesundheit: Kompetenzen. Stärken. Menschen.

[12] Bräutigam C, Enste P, Evans M (2017). Digitalisierung im Krankenhaus: Mehr Technik – bessere Arbeit?.

[13] Brust L, Hartwich NJ, Breidbach C, Antons D (2022). How deep is your work? The day-to-day effects of information and communication technology use on deep work of employees.

[14] Hofstetter S, Lehmann L, Zilezinski M (2022). Vermittlung digitaler Kompetenzen in der Pflegeausbildung – eine Vergleichsanalyse der Rahmenpläne von Bund und Ländern. Bundesgesundheitsbl.

[15] Hübner U, Egbert N, Hackl W (2017). Welche Kernkompetenzen in Pflegeinformatik benötigen Angehörige von Pflegeberufen in den D-A-CH-Ländern? Eine Empfehlung der GMDS, der ÖGPI und der IGPI. Gms Med Inform Epidemiol.

[16] Schüler G, Klaes L, Rommel A (2013). Zukünftiger Qualifikationsbedarf in der Pflege: Ergebnisse und Konsequenzen aus dem BMBF-Forschungsnetz FreQueNz. Bundesgesundheitsbl.

[17] Robert N (2019). How artificial intelligence is changing nursing. Nurs Manage.

[18] Doraiswamy PM, Blease C, Bodner K (2020). Artificial intelligence and the future of psychiatry: insights from a global physician survey. Artif Intell Med.

[19] Carter SM, Rogers W, Win KT (2020). The ethical, legal and social implications of using artificial intelligence systems in breast cancer care. Breast.

[20] Rubin DL (2019). Artificial Intelligence in Imaging: the Radiologist’s Role. J Am Coll Radiol.

[21] Poncette A-S, Glauert DL, Mosch L (2020). Undergraduate medical competencies in digital health and curricular module development: mixed methods study. J Med Internet Res.

[22] Patscha C, Glockner H, Störmer E, Klaffke T (2017) Kompetenz- und Qualifizierungsbedarfe bis 2030. https://www.bmas.de/DE/Service/Publikationen/Broschueren/kompetenz-und-qualifizeirungsbedarfe.html. Zugegriffen: 15. Aug. 2023

[23] Hänold S, Schlee N, Antweiler D, Beckh K (2021). Die Nachvollziehbarkeit von KI-Anwendungen in der Medizin: Eine Betrachtung aus juristischer Perspektive mit Beispielszenarien. MedR.

[24] Boeker M, Klar R (2006). E-Learning in der ärztlichen Aus- und Weiterbildung: Methoden, Ergebnisse, Evaluation. Bundesgesundheitsblatt Gesundheitsforschung Gesundheitsschutz.

[25] Mosch L, Back A, Balzer F (2021). Lernangebote zu Künstlicher Intelligenz in der Medizin.

[26] Jannes M, Friele M, Jannes C (2018). Algorithmen in der digitalen Gesundheitsversorgung: Eine interdisziplinäre Analyse.

[27] (2020) Stanford medicine health trends report: the rise of the data-driven physician. https://med.stanford.edu/content/dam/sm/school/documents/Health-Trends-Report/Stanford%20Medicine%20Health%20Trends%20Report%202020.pdf. Zugegriffen: 15. Aug. 2023

[28] Nickel K, Milde K, Kremer D et al (2022) Bereit für das Smart Hospital? 10.24406/PUBLICA-553

[29] Butcher L (2021). The rise of the healthcare CIO. PLJ.

[30] Shearer C (2000). The CRISP-DM model: the new blueprint for data mining. J Data Warehous.

[31] Rüping S (2015). Big Data in Medizin und Gesundheitswesen. Bundesgesundheitsbl.

[32] Bundschuh BB, Majeed RW, Bürkle T (2011). Quality of human-computer interaction—results of a national usability survey of hospital-IT in Germany. BMC Med Inform Decis Mak.

[33] Seneviratne MG, Li RC, Schreier M (2022). User-centred design for machine learning in health care: a case study from care management. BMJ Health Care Inf.

[34] Werner JA (2022). So krank ist das Krankenhaus: ein Weg zu mehr Menschlichkeit, Qualität und Nachhaltigkeit in der Medizin.

[35] Shamayleh A, Awad M, Farhat J (2020). IoT based predictive maintenance management of medical equipment. J Med Syst.

[36] Europäische Kommission (2021). Vorschlag für eine Verordnung des Europäischen Parlaments und des Rates zur Festlegung Harmonisierter Vorschriften für Künstliche Intelligenz (Gesetz über Künstliche Intelligenz) und zur Änderung bestimmter Rechtsakte der Union.

[37] Cresswell KM, Sheikh A (2015). Health information technology in hospitals: current issues and future trends. Future Hosp J.

[38] Klecun E (2016). Transforming healthcare: policy discourses of IT and patient-centred care. Eur J Inf Syst.

[39] Mirbabaie M, Brünker F, Möllmann Frick NRJ (2022). The rise of artificial intelligence – understanding the AI identity threat at the workplace. Electron Markets.

[40] Reifarth E, Garcia Borrega J, Kochanek M (2023). How to communicate with family members of the critically ill in the intensive care unit: a scoping review. Intensive Crit Care Nurs.

[41] Essen, FOM Bachelor of Arts Pflege & Digitalisierung. https://www.fom.de/die-studiengaenge/gesundheit-und-soziales/bachelor-studiengaenge/pflege-und-digitalisierung.html. Zugegriffen: 15. Aug. 2023

[42] Medizinischer Fakultätentag der Bundesrepublik Deutschland e. V. Nationaler Kompetenzbasierter Lernzielkatalog Medizin. https://nklm.de/zend/menu. Zugegriffen: 27. Juli 2023

[43] Universität Bielefeld Clinician Scientist Programm (CSP). https://www.uni-bielefeld.de/fakultaeten/medizin/karriere/foerderung/clinician-scientist/. Zugegriffen: 15. Aug. 2023

[44] TU Dresden Curriculum Clinicum Digitale. https://tu-dresden.de/ing/studium/termine/clinicum-digitale-springschool-fuer-studierende-der-informatik-medizin. Zugegriffen: 15. Aug. 2023

[45] RWTH Aachen Medical data science M.sc. https://www.rwth-aachen.de/cms/root/studium/Vor-dem-Studium/Studiengaenge/Liste-Aktuelle-Studiengaenge/Studiengangbeschreibung/~eqyqp/Medical-Data-Science-M-Sc/. Zugegriffen: 15. Aug. 2023

[46] Hinding B, Gornostayeva M, Lux R et al (2020) Kommunikative Kompetenzen von Ärztinnen und Ärzten. https://www.impp.de/files/PDF/BMG-Berichte/IMPP-Leitfaden-Kommunikative-Kompetenzen_komprimiert.pdf. Zugegriffen: 15. Aug. 2023

[47] Bessen J (2018). Artificial intelligence and jobs: the role of demand. The economics of artificial intelligence: an agenda.

[48] Weber M, Kaiser F (2022). Die digitale Transformation im Krankenhausalltag. Digitalstrategie im Krankenhaus: Einführung und Umsetzung von Datenkompetenz und Compliance.

[49] Kostick-Quenet KM, Gerke S (2022). AI in the hands of imperfect users. Npj Digit Med.

[50] Singhal K, Azizi S, Tu T (2023). Large language models encode clinical knowledge. Nature.

