# Supplementary material for: Use of AI-based applications by hospital staff: task profiles and qualification requirements
Source: Bundesgesundheitsblatt Gesundheitsforschung Gesundheitsschutz. 2023 Nov 30;67(1):66–75. [Article in German] doi: 10.1007/s00103-023-03817-x (PMC10776476; doi:10.1007/s00103-023-03817-x)
Supplement: Supplementary file 1 [file 103_2023_3817_MOESM1_ESM.pdf]

Einsatz von KI-basierten Anwendungen durch Krankenhauspersonal: Aufgabenprofile und Qualifizierungsbedarfe

Bundesgesundheitsblatt Ausgabe 01/2024

Onlinematerial Nr. 1: Literaturliste

- Alt, Christina, Corinna von Au, und Marek Bartzik. 2021. Future Skills in Medizin und Gesundheit: Kompetenzen. Stärken. Menschen. Herausgegeben von David Matusiewicz, Jochen A. Werner, und Martin Hirsch. Berlin: Medizinisch Wissenschaftliche Verlagsgesellschaft.
- Amisha, Paras Malik, Monika Pathania, und VyasKumar Rathaur. 2019. „Overview of Artificial Intelligence in Medicine“. Journal of Family Medicine and Primary Care 8 (7): 2328. https://doi.org/10.4103/jfmpc.jfmpc_440_19.
- Anhäuser, Marcus, Holger Wormer, Astrid Viciano, und Wiebke Rögener. 2021. „Ein modulares Modell zur Qualitätssicherung im Medizin- und Ernährungsjournalismus“. Bundesgesundheitsblatt - Gesundheitsforschung - Gesundheitsschutz 64 (1): 12–20. https://doi.org/10.1007/s00103-020-03254-0.
- Bittlingmayer, Uwe H., Kevin Dadaczynski, Diana Sahrai, Stephan van den Broucke, und Orkan Okan. 2020. „Digitale Gesundheitskompetenz – Konzeptionelle Verortung, Erfassung und Förderung mit Fokus auf Kinder und Jugendliche“. Bundesgesundheitsblatt - Gesundheitsforschung - Gesundheitsschutz 63 (2): 176–84. https://doi.org/10.1007/s00103-019-03087-6.
- Blease, Charlotte, Michael H. Bernstein, Jens Gaab, Ted J. Kaptchuk, Joe Kossowsky, Kenneth D. Mandl, Roger B. Davis, und Catherine M. DesRoches. 2018. „Computerization and the Future of Primary Care: A Survey of General Practitioners in the UK“. Herausgegeben von Lars-Peter Kamolz. PLOS ONE 13 (12): e0207418. https://doi.org/10.1371/journal.pone.0207418.
- Boeing, Niels, Klaus Burmeister, Andreas Neef, Ben Rodenhäuser, und Willi Schroll. 2014. „CONNECTED REALITY 2025 Die nächste Welle der digitalen Transformation“. Z_punkt GmbH.
- Boeker, M., und R. Klar. 2006. „E-Learning in der ärztlichen Aus- und Weiterbildung: Methoden, Ergebnisse, Evaluation“. Bundesgesundheitsblatt - Gesundheitsforschung - Gesundheitsschutz 49 (5): 405–11. https://doi.org/10.1007/s00103-006-1259-y.
- Bräutigam, Christoph, Peter Enste, Michaela Evans, Josef Hilbert, Sebastian Merkel, und Fikret Öz. 2017. „Digitalisierung im Krankenhaus: Mehr Technik – bessere Arbeit?“ Hans-Böckler-Stiftung.
- Brockmann, Dirk. 2020. „Digitale Epidemiologie“. Bundesgesundheitsblatt - Gesundheitsforschung - Gesundheitsschutz 63 (2): 166–75. https://doi.org/10.1007/s00103-019-03080-z.
- Broich, Karl, Wiebke Löbker, und Wolfgang Lauer. 2021. „Beitrag des BfArM zur Potenzialentfaltung der Digitalisierung im Gesundheitswesen – digital readiness@BfArM“. Bundesgesundheitsblatt - Gesundheitsforschung - Gesundheitsschutz 64 (10): 1292–97. https://doi.org/10.1007/s00103-021-03417-7.

- Brust, Leona, Nicole Janine Hartwich, Christoph Breidbach, und David Antons. 2022. „How Deep is your Work? The Day-to-Day Effects of Information and Communication Technology Use on Deep Work of Employees“, ICIS 2022 Proceedings, . https://aisel.aisnet.org/icis2022/is_futureofwork/is_futureofwork/13.
- Butcher, Lola. 2021. „The Rise of the Healthcare CIO“. Physician Leadership Journal 8 (3): 20–22.
- Carter, Stacy M., Wendy Rogers, Khin Than Win, Helen Frazer, Bernadette Richards, und Nehmat Houssami. 2020. „The Ethical, Legal and Social Implications of Using Artificial Intelligence Systems in Breast Cancer Care“. The Breast 49 (Februar): 25–32. https://doi.org/10.1016/j.breast.2019.10.001.
- Chew, Han Shi Jocelyn, und Palakorn Achananuparp. 2022. „Perceptions and Needs of Artificial Intelligence in Health Care to Increase Adoption: Scoping Review“. Journal of Medical Internet Research 24 (1): e32939. https://doi.org/10.2196/32939.
- Couffinhal, Agnès. 2017. „Tackling Wasteful Spending on Health“. OECD Publishing. oe.cd/tackling-wasteful-spending-on-health.
- Dadaczynski, Kevin, Orkan Okan, Melanie Messer, Angela Y M Leung, Rafaela Rosário, Emily Darlington, und Katharina Rathmann. 2021. „Digital Health Literacy and Web-Based Information-Seeking Behaviors of University Students in Germany During the COVID-19 Pandemic: Cross-Sectional Survey Study“. Journal of Medical Internet Research 23 (1): e24097. https://doi.org/10.2196/24097.
- Daum, Mario. 2017. „Digitalisierung und Technisierung der Pflege in Deutschland“. INPUT Consulting gGmbH.
- Davenport, Thomas, und Ravi Kalakota. 2019. „The Potential for Artificial Intelligence in Healthcare“. Future Healthcare Journal 6 (2): 94–98. https://doi.org/10.7861/futurehosp.6-2-94.
- Demner-Fushman, Dina, Wendy W. Chapman, und Clement J. McDonald. 2009. „What Can Natural Language Processing Do for Clinical Decision Support?“ Journal of Biomedical Informatics 42 (5): 760–72. https://doi.org/10.1016/j.jbi.2009.08.007.
- Doraiswamy, P. Murali, Charlotte Blease, und Kaylee Bodner. 2020. „Artificial Intelligence and the Future of Psychiatry: Insights from a Global Physician Survey“. Artificial Intelligence in Medicine 102 (Januar): 101753. https://doi.org/10.1016/j.artmed.2019.101753.
- Fliegenschmidt, Janis, Nikolai Hulde, Maria G. Preising, Silvia Ruggeri, Ralph Szymanowski, Laurent Meesseman, Hong Sun, und Vera von Dossow. 2021. „Artificial Intelligence Predicts Delirium Following Cardiac Surgery: A Case Study“. Journal of Clinical Anesthesia 75 (Dezember): 110473. https://doi.org/10.1016/j.jclinane.2021.110473.
- Gehring, H., K. Rackebrandt, und M. Imhoff. 2018. „E-Health und die Realität – was sehen wir heute schon in der Klinik?“ Bundesgesundheitsblatt - Gesundheitsforschung - Gesundheitsschutz 61 (3): 252–62. https://doi.org/10.1007/s00103-018-2690-6.
- Habli, Ibrahim, Tom Lawton, und Zoe Porter. 2020. „Artificial intelligence in health care: accountability and safety“. Bulletin of the World Health Organization 98 (4): 251–56. https://doi.org/10.2471/BLT.19.237487.
- Hänold, Stefanie, Nelli Schlee, Dario Antweiler, und Katharina Beckh. 2021. „Die Nachvollziehbarkeit von KI-Anwendungen in der Medizin: Eine Betrachtung aus juristischer Perspektive mit Beispielszenarien“. Medizinrecht 39 (6): 516–23. https://doi.org/10.1007/s00350-021-5901-3.

- Hinding, Barbara, Maryna Gornostayeva, Richard Lux, Christian Brünahl, Holger Buggenhagen, Nadine Gronewold, Anke Hollinderbäumer, Kirsten Reschke, Jobst-Hendrik Schultz, und Jana Jünger. 2020. „Kommunikative Kompetenzen von Ärztinnen und Ärzten“. Institut für medizinische und pharmazeutische Prüfungsfragen.
- Hofstetter, Sebastian, Lisa Lehmann, Max Zilezinski, Jenny-Victoria Steindorff, Patrick Jahn, und Denny Paulicke. 2022. „Vermittlung digitaler Kompetenzen in der Pflegeausbildung – eine Vergleichsanalyse der Rahmenpläne von Bund und Ländern“. Bundesgesundheitsblatt - Gesundheitsforschung - Gesundheitsschutz 65 (9): 891–99. https://doi.org/10.1007/s00103-022-03575-2.
- Hübner, Ursula, Nicole Egbert, Werner Hackl, Martin Lysser, Georg Schulte, Johannes Thye, und Elske Ammenwerth. 2017. „Welche Kernkompetenzen in Pflegeinformatik benötigen Angehörige von Pflegeberufen in den D-A-CH-Ländern? Eine Empfehlung der GMDS, der ÖGPI und der IGPI“. Text/html. GMS Medizinische Informatik, Juli, Biometrie und Epidemiologie; 13(1):Doc02. https://doi.org/10.3205/MIBE000169.
- Huisman, Merel, Erik Ranschaert, William Parker, Domenico Mastrodicasa, Martin Koci, Daniel Pinto de Santos, Francesca Coppola, u. a. 2021. „An International Survey on AI in Radiology in 1041 Radiologists and Radiology Residents Part 2: Expectations, Hurdles to Implementation, and Education“. European Radiology 31 (11): 8797–8806. https://doi.org/10.1007/s00330-021-07782-4.
- Jannes, Marc, Minou Friele, Christiane Jannes, Christiane Woopen, und Bertelsmann Stiftung. 2018. „Algorithmen in der digitalen Gesundheitsversorgung: Eine interdisziplinäre Analyse“. https://doi.org/10.11586/2019053.
- Jiang, Fei, Yong Jiang, Hui Zhi, Yi Dong, Hao Li, Sufeng Ma, Yilong Wang, Qiang Dong, Haipeng Shen, und Yongjun Wang. 2017. „Artificial Intelligence in Healthcare: Past, Present and Future“. Stroke and Vascular Neurology 2 (4): 230–43. https://doi.org/10.1136/svn-2017-000101.
- Jünger, Jana. 2020. „Sachbericht: Kommunikative Kompetenzen von Ärztinnen und Ärzten“. IMPP - Institut für medizinische und pharmazeutische Prüfungsfragen.
- Koch, Klaus. 2015. „Deutsches Netzwerk Evidenzbasierte Medizin. Gute Praxis Gesundheitsinformation.“ http://www.ebm-netzwerk.de/gpgi.
- Kreyenschulte, Thea, und Sabine Bohnet-Joschko. 2022. „Erwartungen der Generation Y an digitale Gesundheitsinnovationen“. Bundesgesundheitsblatt - Gesundheitsforschung - Gesundheitsschutz 65 (9): 881–90. https://doi.org/10.1007/s00103-022-03567-2.
- Kuhn, Sebastian, D Ammann, I Cichon, J Ehlers, S Guttormsen, M Hülsken-Giesler, S Kaap-Fröhlich, u. a. 2019. „Careum working paper 8 - short version: «Wie revolutioniert die digitale Transformation die Bildung der Berufe im Gesundheitswesen?»“. Careum Stiftung. www.careum.ch/workingpaper8-kurz.
- Kunz, Thomas, Benjamin Lange, und Annika Selzer. 2020. „Datenschutz und Datensicherheit in Digital Public Health“. Bundesgesundheitsblatt - Gesundheitsforschung - Gesundheitsschutz 63 (2): 206–14. https://doi.org/10.1007/s00103-019-03083-w.
- Langewitz, W. 2012. „Zur Erlernbarkeit der Arzt-Patienten-Kommunikation in der Medizinischen Ausbildung“. Bundesgesundheitsblatt - Gesundheitsforschung - Gesundheitsschutz 55 (9): 1176–82. https://doi.org/10.1007/s00103-012-1533-0.
- Marckmann, Georg. 2020. „Ethische Fragen von Digital Public Health“. Bundesgesundheitsblatt - Gesundheitsforschung - Gesundheitsschutz 63 (2): 199–205. https://doi.org/10.1007/s00103-019-03091-w.

- McCoy, Liam G., Sujay Nagaraj, Felipe Morgado, Vinyas Harish, Sunit Das, und Leo Anthony Celi. 2020. „What Do Medical Students Actually Need to Know about Artificial Intelligence?“ Npj Digital Medicine 3 (1): 86. https://doi.org/10.1038/s41746-020-0294-7.
- Meskó, Bertalan, Gergely Hetényi, und Zsuzsanna Győrffy. 2018. „Will Artificial Intelligence Solve the Human Resource Crisis in Healthcare?“ BMC Health Services Research 18 (1): 545. https://doi.org/10.1186/s12913-018-3359-4.
- Meyer, Melanie A. 2019. „Healthcare Data Scientist Qualifications, Skills, and Job Focus: A Content Analysis of Job Postings“. Journal of the American Medical Informatics Association 26 (5): 383–91. https://doi.org/10.1093/jamia/ocy181.
- Miotto, Riccardo, Fei Wang, Shuang Wang, Xiaoqian Jiang, und Joel T Dudley. 2018. „Deep Learning for Healthcare: Review, Opportunities and Challenges“. Briefings in Bioinformatics 19 (6): 1236–46. https://doi.org/10.1093/bib/bbx044.
- Mohr, Jutta, Riedlinger, Isabelle, und Reiber, Karin. 2020. „Die Bedeutung der Digitalisierung in der Neuausrichtung der pflegerischen Ausbildung – Herausforderungen für die berufliche Pflege im Kontext der Fachkräftesicherung“, September. https://doi.org/10.25656/01:20661.
- Mosch, Lina, Daniel Fürstenau, Jenny Brandt, Jasper Wagnitz, Sophie AI Klopfenstein, Akira-Sebastian Poncette, und Felix Balzer. 2022. „The Medical Profession Transformed by Artificial Intelligence: Qualitative Study“. DIGITAL HEALTH 8 (Januar): 205520762211439. https://doi.org/10.1177/20552076221143903.
- Nickel, Kilian, Katharina Milde, Dustin Kremer, Christine Malich, Dario Antweiler, Thomas Reibel, Florian Jovy-Klein, Jil Sander, Dominik Bures, und Anke Diehl. 2022. „Bereit für das Smart Hospital?“, November. https://doi.org/10.24406/PUBLICA-553.
- Norman, Cameron D, und Harvey A Skinner. 2006. „EHealth Literacy: Essential Skills for Consumer Health in a Networked World“. Journal of Medical Internet Research 8 (2): e9. https://doi.org/10.2196/jmir.8.2.e9.
- „Nutzertestung von Gesundheitsinformationen des IQWiG durch sozial benachteiligte Personen“. 2014.
- Pandey, Babita, Devendra Kumar Pandey, Brijendra Pratap Mishra, und Wasiur Rhmann. 2022. „A Comprehensive Survey of Deep Learning in the Field of Medical Imaging and Medical Natural Language Processing: Challenges and Research Directions“. Journal of King Saud University - Computer and Information Sciences 34 (8): 5083–99. https://doi.org/10.1016/j.jksuci.2021.01.007.
- Papp-Zipernovszky, Orsolya, Mária Dóra Horváth, Peter J. Schulz, und Márta Csabai. 2021. „Generation Gaps in Digital Health Literacy and Their Impact on Health Information Seeking Behavior and Health Empowerment in Hungary“. Frontiers in Public Health 9 (Mai): 635943. https://doi.org/10.3389/fpubh.2021.635943.
- Paranjape, Ketan, Michiel Schinkel, Rishi Nannan Panday, Josip Car, und Prabath Nanayakkara. 2019. „Introducing Artificial Intelligence Training in Medical Education“. JMIR Medical Education 5 (2): e16048. https://doi.org/10.2196/16048.
- Patscha, Cornelius, Holger Glockner, Eckhard Störmer, und Thomas Klaffke. 2017. „Kompetenz- und Qualifizierungsbedarfe bis 2030“. Bundesministerium für Arbeit und Soziales.
- Peete, Rashida, Kasia Majowski, Laura Lauer, und Annie Jay. 2019. „Artificial Intelligence in Healthcare“. In Artificial Intelligence and Machine Learning for Business for Non-Engineers, herausgegeben von Frank M. Groom und Stephan S. Jones, 1. Aufl., 89–101. CRC Press. https://doi.org/10.1201/9780367821654-8.

– Peissner, Matthias, Falko Kötter, und Helmut Zaiser. 2019. „Künstliche Intelligenz – Anwendungsperspektiven für Arbeit und Qualifizierung“, BWP, . https://www.bwp-zeitschrift.de/dienst/publikationen/ihbb/10023.
– Pesapane, Filippo, Caterina Volonté, Marina Codari, und Francesco Sardanelli. 2018. „Artificial Intelligence as a Medical Device in Radiology: Ethical and Regulatory Issues in Europe and the United States“. Insights into Imaging 9 (5): 745–53. https://doi.org/10.1007/s13244-018-0645-y.
– Poncette, Akira-Sebastian, Daniel Leon Glauert, Lina Mosch, Katarina Braune, Felix Balzer, und David Alexander Back. 2020. „Undergraduate Medical Competencies in Digital Health and Curricular Module Development: Mixed Methods Study“. Journal of Medical Internet Research 22 (10): e22161. https://doi.org/10.2196/22161.
– Rajpurkar, Pranav, Emma Chen, Oishi Banerjee, und Eric J. Topol. 2022. „AI in Health and Medicine“. Nature Medicine 28 (1): 31–38. https://doi.org/10.1038/s41591-021-01614-0.
– Reifarth, Eyleen, Jorge Garcia Borrega, und Matthias Kochanek. 2023. „How to Communicate with Family Members of the Critically Ill in the Intensive Care Unit: A Scoping Review“. Intensive and Critical Care Nursing 74 (Februar): 103328. https://doi.org/10.1016/j.iccn.2022.103328.
– Reixach, Elisenda, Erik Andrés, Josuè Sallent Ribes, Montserrat Gea-Sánchez, Alícia Àvila López, Bea Cruañas, Anna González Abad, u. a. 2022. „Measuring the Digital Skills of Catalan Health Care Professionals as a Key Step Toward a Strategic Training Plan: Digital Competence Test Validation Study“. Journal of Medical Internet Research 24 (11): e38347. https://doi.org/10.2196/38347.
– Reyes-Ortiz, Jose A., Beatriz A. Gonzalez-Beltran, und Lizbeth Gallardo-Lopez. 2015. „Clinical Decision Support Systems: A Survey of NLP-Based Approaches from Unstructured Data“. In 2015 26th International Workshop on Database and Expert Systems Applications (DEXA), 163–67. Valencia: IEEE. https://doi.org/10.1109/DEXA.2015.47.
– Robert, Nancy. 2019. „How Artificial Intelligence Is Changing Nursing“. Nursing Management 50 (9): 30–39. https://doi.org/10.1097/01.NUMA.0000578988.56622.21.
– Rubin, Daniel L. 2019. „Artificial Intelligence in Imaging: The Radiologist’s Role“. Journal of the American College of Radiology 16 (9): 1309–17. https://doi.org/10.1016/j.jacr.2019.05.036.
– Rüping, Stefan. 2015. „Big Data in Medizin und Gesundheitswesen“. Bundesgesundheitsblatt - Gesundheitsforschung - Gesundheitsschutz 58 (8): 794–98. https://doi.org/10.1007/s00103-015-2181-y.
– Salge, Torsten Oliver, David Antons, Michael Barrett, Rajiv Kohli, Eivor Oborn, und Stavros Polykarpou. 2022. „How IT Investments Help Hospitals Gain and Sustain Reputation in the Media: The Role of Signaling and Framing“. Information Systems Research 33 (1): 110–30. https://doi.org/10.1287/isre.2021.1021.
– Schaeffer, D, K Hurrelmann, U Bauer, und K Kolpatzik. 2018. „Nationaler Aktionsplan Gesundheitskompetenz. Die Gesundheitskompetenz in Deutschland stärken.“

- Schaffter, Thomas, Diana S. M. Buist, Christoph I. Lee, Yaroslav Nikulin, Dezso Ribli, Yuanfang Guan, William Lotter, u. a. 2020. „Evaluation of Combined Artificial Intelligence and Radiologist Assessment to Interpret Screening Mammograms". JAMA Network Open 3 (3): e200265. https://doi.org/10.1001/jamanetworkopen.2020.0265.
- Schneider, Diana, Arne Sonar, und Karsten Weber. 2022. „Zwischen Automatisierung und ethischem Anspruch – Disruptive Effekte des KI-Einsatzes in und auf Professionen der Gesundheitsversorgung". In Künstliche Intelligenz im Gesundheitswesen, herausgegeben von Mario A. Pfannstiel, 325–48. Wiesbaden: Springer Fachmedien Wiesbaden. https://doi.org/10.1007/978-3-658-33597-7_14.
- Schüler, G., L. Klaes, A. Rommel, H. Schröder, und T. Köhler. 2013. „Zukünftiger Qualifikationsbedarf in der Pflege: Ergebnisse und Konsequenzen aus dem BMBF-Forschungsnetz FreQueNz". Bundesgesundheitsblatt - Gesundheitsforschung - Gesundheitsschutz 56 (8): 1135–44. https://doi.org/10.1007/s00103-013-1754-x.
- Sendak, Mark P, William Ratliff, Dina Sarro, Elizabeth Alderton, Joseph Futoma, Michael Gao, Marshall Nichols, u. a. 2020. „Real-World Integration of a Sepsis Deep Learning Technology Into Routine Clinical Care: Implementation Study". JMIR Medical Informatics 8 (7): e15182. https://doi.org/10.2196/15182.
- Shamayleh, Abdulrahim, Mahmoud Awad, und Jumana Farhat. 2020. „IoT Based Predictive Maintenance Management of Medical Equipment". Journal of Medical Systems 44 (4): 72. https://doi.org/10.1007/s10916-020-1534-8.
- Shearer, C. 2000. „The CRISP-DM Model: The New Blueprint for Data Mining". Journal of Data Warehousing, Nr. 5.
- „Stanford Medicine 2020 Health Trends Report: The Rise of the Data-Driven Physician". 2020. https://med.stanford.edu/news/all-news/2020/01/health-trends-report-spotlights-rise-of-data-driven-physician.html.
- Stead, Susan, Lina Vogt, David Antons, Oliver Salge, Judith Gecht, Martin Klasen, und Sasa Sopka. 2023. „Hospital Resource Endowments and Nosocomial Infections: Longitudinal Evidence from the English National Health System on Clostridioides Difficile between 2011 and 2019". Journal of Hospital Infection, Februar, S0195670123000385. https://doi.org/10.1016/j.jhin.2023.01.014.
- Tang, Xiaoli, Brian Wang, und Yi Rong. 2018. „Artificial Intelligence Will Reduce the Need for Clinical Medical Physicists". Journal of Applied Clinical Medical Physics 19 (1): 6–9. https://doi.org/10.1002/acm2.12244.
- Tekkeşin, Ahmet İlker. 2019. „Artificial Intelligence in Healthcare: Past, Present and Future". The Anatolian Journal of Cardiology. https://doi.org/10.14744/AnatolJCardiol.2019.28661.
- Thompson, Reid F., Gilmer Valdes, Clifton D. Fuller, Colin M. Carpenter, Olivier Morin, Sanjay Aneja, William D. Lindsay, u. a. 2018. „Artificial Intelligence in Radiation Oncology: A Specialty-Wide Disruptive Transformation?" Radiotherapy and Oncology 129 (3): 421–26. https://doi.org/10.1016/j.radonc.2018.05.030.
- Tong, Siliang, Nan Jia, Xueming Luo, und Zheng Fang. 2021. „The Janus Face of Artificial Intelligence Feedback: Deployment versus Disclosure Effects on Employee Performance". Strategic Management Journal 42 (9): 1600–1631. https://doi.org/10.1002/smj.3322.
- Werner, Jochen A. 2022. So krank ist das Krankenhaus: ein Weg zu mehr Menschlichkeit, Qualität und Nachhaltigkeit in der Medizin. 1. Auflage. Essen: Klartext Verlag.

– Werner, Jochen A., Michael Forsting, Thorsten Kaatze, und Andrea Schmidt-Rumposch. 2020. Smart Hospital: digitale und empathische Zukunftsmedizin. Berlin: Medizinisch WissenschaftlicheVerlagsgesellschaft.
– Werner, Jochen A., Thorsten Kaatze, und Andrea Schmidt-Rumposch. 2022. Green Hospital: Nachhaltigkeit und Ressourcenschonung im Krankenhaus. Berlin: Medizinisch Wissenschaftliche Verlagsgesellschaft.
– Zeng, Fengyi, Xiaowen Liang, und Zhiyi Chen. 2020. „New Roles for Clinicians in the Age of Artificial Intelligence“. BIO Integration 1 (3): 113–17. https://doi.org/10.15212/bioi-2020-0014.
– Zens, Maria, Yvette Shajanian Zarneh, Jürgen Dolle, und Freia De Bock. 2020. „Digital Public Health – Hebel für Capacity Building in der kommunalen Gesundheitsförderung: Ausgangslage, Entwicklungsfragen, TEAviisari als modellhafte Implementierung“. Bundesgesundheitsblatt - Gesundheitsforschung - Gesundheitsschutz 63 (6): 729–40. https://doi.org/10.1007/s00103-020-03148-1.

Onlinematerial Nr. 2: Interview-Leitfaden

Rahmen

- Ziel:
  - Identifikation Status Quo der KI-Qualifikation im KH-Personal
  - Abfrage nach Qualifizierungsbedarfen
  - Feedbackabfrage zu Aufgabenprofilen und Qualifizierungsbedarfen
- Zielgruppe:
  - Krankenhauspersonal, welches bereits jetzt oder zukünftig mit KI-Systemen in Kontakt stehen
- Rahmen: 90 Minuten Interview

Inhalte

#1 - Einleitung

- Guten Tag, vielen Dank für Ihre Zeit für das Gespräch.
- Mein Name ist XY, ich bin Wissenschaftler*in am Fraunhofer IAIS. Das ist meine Kolleg*in XY. Wir arbeiten im Bereich der Informatik an den Themen Data Science und Künstliche Intelligenz.
- Wir führen diese Interviews im Rahmen eines Forschungsprojektes durch und möchten mit Ihnen gerne über das Thema „KI-Systeme und Qualifizierung von Mitarbeiterinnen“ sprechen.
- Wir machen uns Notizen und fertigen ein anonymisiertes Protokoll an. Gemeinsam mit anderen Interviews werten wir alle Antworten anonym aus und fassen sie zusammen. Falls gewünscht, können wir es Ihnen gerne zukommen lassen.
- Sind Sie damit einverstanden? Haben Sie noch Fragen?
- Würden Sie sich bitte kurz vorstellen

#2 - Einordnung Projektrahmen

(1) Das SmartHospital.NRW-Projekt hat als Ziel die „Entwicklung eines smarten Modells zur Transformation von Krankenhäusern (Hospitals) in NRW mittels innovativer Use Cases basierend auf Künstlicher Intelligenz (KI) in der Medizin“.

(2) Es geht also um die erfolgreiche Umsetzung von KI-Systemen im Krankenhaus.

(3) Wir verstehen unter Künstlicher Intelligenz alle Software-Systeme, die einen lernenden Charakter haben und eine Aufgabe nachahmen, die ein Mensch bereits durchführen kann. Dazu zählen beispielsweise Bildverarbeitung, Spracherkennung, Robotik oder Identifikation komplexer Zusammenhänge in großen Datenmengen.

(4) Bei einer erfolgreichen Umsetzung von KI-Systemen ist ein wesentlicher Bestandteil, dass das Personal im Krankenhaus die erforderlichen Qualifikationen für den Einsatz besitzt.

(5) Unter Qualifikationen verstehen wir alle Fähigkeiten oder Skills, die eine Person benötigt, um ein System in ihren Tätigkeiten einzusetzen.

(6) Wir möchten heute herausfinden, welche Qualifikationen das sind und welche Personen im Krankenhaus diese erwerben sollten.

#3 - Identifikation Status Quo

(7) In welchem Bereich arbeiten Sie? Welche Rollen nehmen Sie dort ein?
- a) Was sind Ihre Haupttätigkeiten in Ihrem Bereich? Haben Sie noch weitere Tätigkeiten außerhalb Ihres Bereichs?

(8) Verwenden Sie selbst KI-Systeme in Ihrer Arbeit?
- a) Für welchen Prozess/mit welchem Ziel?
- b) In welcher Regelmäßigkeit?
- c) Welche anderen Personen sind dabei involviert?
- d) Wie wurden Sie auf die Verwendung vorbereitet? Wie sähe eine ideale Vorbereitung aus?

(9) Kennen Sie Einsatzorte im Klinikum für KI-Systeme? (z.B. Spracherkennung, OP-Robotik, Bildauswertung, Entscheidungsunterstützung)
- a) Für welche Prozesse werden diese Systeme eingesetzt?
- b) Welche Personen setzen diese Systeme ein?
- c) Wie wurden die Personen für diese Aufgabe vorbereitet? (Schulungen, Seminare, Onboardings, Lernplattformen, Lernvideos)
- d) Wieviel Zeit wird für Qualifizierungsbedarfe beansprucht?
- e) Welche Qualifikationen bringen die Personen für die Verwendung des Systems mit?
- f) Welche Systeme haben besonders hohe Komplexität bei der Bedienung?

(10) Welche Personen in Ihrem Krankenhaus haben die Aufgabe eines Transfers zwischen Medizin und Technologie?

(11) Gibt es aktuell Ausschreibungen für Personal, die KI-Systeme einsetzen sollen?

#4 - Abfrage nach Qualifizierungsbedarf

(12) Welche KI-Systeme werden in Zukunft vermehrt eingesetzt werden?

(13) Welche Bereiche werden besonders von dem erhöhten Einsatz geprägt werden?

(14) Welche Personen werden besonders von dem erhöhten Einsatz von KI in ihrer Tätigkeit betroffen sein? (1 = kaum, 5 = sehr stark)

a) Ärztliches Personal
b) Pflegerisches Personal
c) Medizin-Controlling
d) Patientenmanagement
e) IT/Verwaltung
f) Labor
g) Apotheke
h) andere Bereiche

(15) Welche Skills oder Fähigkeiten werden je Berufsgruppe durch den erhöhten Einsatz von KI im Klinikum in Zukunft notwendig werden?

a) Ärztliches Personal
b) Pflegerisches Personal
c) Medizin-Controlling
d) Patientenmanagement
e) IT/Verwaltung
f) Labor
g) Apotheke
h) andere Bereiche

(16) Welche neuen Aufgabenprofile könnte es durch den erhöhten Einsatz von KI in Zukunft geben?

(17) Welche Herausforderungen für das Personal wird es durch den erhöhten Einsatz von KI in Zukunft geben? (z.B.: Datenbias, Fehlererkennung, Vertrauen, Automation Bias)

(18) Wie könnte man diese Herausforderungen bewältigen?

(19) Wie können die betroffenen Personen diese Skills und Fähigkeiten erwerben?

(20) Welche geschlechter-spezifischen Unterschiede gibt es in den genannten Aspekten?

#5 - Feedbackabfrage zu Aufgabenprofilen und Qualifizierungsbedarfen

(21) Wir haben folgende Aufgabenprofile entworfen und würden dazu gerne Ihr Feedback erfassen. Bitte geben Sie jeweils an, ob Sie es als wahrscheinlich einschätzen, dass es in Zukunft ein solches Aufgabenprofil in Ihrem Krankenhaus geben wird. (1 = sehr unwahrscheinlich, 5 sehr wahrscheinlich)

- Liste der vorhandenen Aufgabenprofile

(22) Wir haben folgende Qualifizierungsmaßnahmen entworfen und würden dazu gerne Ihr Feedback erfassen. Bitte geben Sie jeweils an, ob Sie persönlich an einer solchen Maßnahme teilnehmen würden (1 = sehr unwahrscheinlich, 5 sehr wahrscheinlich) und ob Sie diese Maßnahme als für notwendig im Allgemeinen halten (1 = nicht notwendig, 5 = sehr notwendig)

- Liste der vorhandenen Qualifizierungsmaßnahmen

(23) Haben Sie noch weitere Ideen für Qualifizierungsmaßnahmen?

#6 - Abschluss

(24) Haben Sie noch Fragen an uns? Möchten Sie uns noch etwas mitteilen?

Onlinematerial Nr. 3: Liste von Interviews

No.	Fachbereiche	Institution
1	Pflege	A
2	Pflege	A
3	IT	A
4	Medizin-Controlling x2	A
5	Leitung / Ärzteschaft	B
6	Personalplanung	B
7	Leitung / Ärzteschaft	A
8	OP-Bereich	A
9	Leitung / IT	A
10	Ärzteschaft	B

Onlinematerial Nr. 4: Liste von KI Use Cases

No.	Name	Quelle des Use Cases	Link
1	Semi-automatische Extraktion von Informationen aus Arztbriefen zur Überführung in strukturierte Form	SmartHospital.NRW	https://smarthospital.nrw/
2	Semi-automatisierte Erstellung von Entlassbriefen	SmartHospital.NRW	https://smarthospital.nrw/

3	Sprach- und Dialogsystem im Patient*innenzimmer	SmartHospital.NRW	https://smarthospital.nrw/
4	Sprachsteuerung Angiographie	SmartHospital.NRW	https://smarthospital.nrw/
5	COPD-Monitoring	SmartHospital.NRW	https://smarthospital.nrw/
6	Pneumonie Prävention	SmartHospital.NRW	https://smarthospital.nrw/
7	Sturz-Prophylaxe	SmartHospital.NRW	https://smarthospital.nrw/
8	Dekubitus-Prophylaxe	SmartHospital.NRW	https://smarthospital.nrw/
9	Schmerzmanagement	SmartHospital.NRW	https://smarthospital.nrw/
10	Delirium-Prophylaxe	SmartHospital.NRW	https://smarthospital.nrw/
11	Kontinenzmanagement	SmartHospital.NRW	https://smarthospital.nrw/
12	Trajektorien Klassifikation	LOTTE	https://www.iais.fraunhofer.de/lotte
13	OP Risikoabschätzung (im Schockraum)	LOTTE	https://www.iais.fraunhofer.de/lotte
14	(semi) automatische Dokumentation Schockraum	LOTTE	https://www.iais.fraunhofer.de/lotte
15	Intelligentes Leitlinien Interface	LOTTE	https://www.iais.fraunhofer.de/lotte
16	Literature Mining	LOTTE	https://www.iais.fraunhofer.de/lotte
17	Intelligente Alarmierungskette	LOTTE	https://www.iais.fraunhofer.de/lotte
18	Rightcoding	GSG Rico	https://gsg-online.com/fallbegleitende-kodierung/
19	Assistierte Klinische Dokumentation	Externe Projekte	
20	Dokumentation Anamnese	Externe Projekte	
21	Spracherkennung für Klinische Dokumentation	TraumAInterfaces	https://www.time.rwth-aachen.de/cms/TIME/Forschung/Healthcare-Innovation-Lab/Projekte/~mqhlw/TraumAInterfaces/
22	Workflow Optimization	AutoPiLoT	https://www.fh-dortmund.de/projekte/autopilot.php
23	Automatisierung von Routinen	AutoPiLoT	https://www.fh-dortmund.de/projekte/autopilot.php
24	Diagnose von selten Erkrankungen	WisPerMed	https://wispermed.com/
25	Radiologie - Gehirn	Externe Projekte	
26	Radiologie - Lungen	Externe Projekte	
27	Radiologie - Herz	Externe Projekte	
28	Pflegeassessment Support	Externe Projekte	
29	Digitaler Zwilling	MED²ICIN	https://websites.fraunhofer.de/med2icin/

30	Robotik im OP	Externe Projekte	
31	Automatisierte Medikamentenbeschaffung	Externe Projekte	
32	Logistik - Patiententransport	Externe Projekte	
33	Green Hospital	Externe Projekte	
34	Roboter Unterstützung für Routinetätigkeiten	RoReBo	https://www.ipa.fraunhofer.de/de/presse/presseinformationen/schluesseltechnologien-fuer-reinigungsroboter-im-gesundheitswesen.html
35	Desinfektion von Räumen mithilfe von Robotern	RoReBo	https://www.ipa.fraunhofer.de/de/presse/presseinformationen/schluesseltechnologien-fuer-reinigungsroboter-im-gesundheitswesen.html
36	Pflegerobotik zur physischen Unterstüzung	Elevon	https://www.ipa.fraunhofer.de/de/referenzprojekte/Elevon.html
37	Pflegerobotik für sozialen Umgang	ASARob	https://www.ipa.fraunhofer.de/de/referenzprojekte/ASARoB.html
38	KI gestütze Laboranalysen	WisPerMed	https://wispermed.com/

Onlinematerial Nr. 5: Erfassung von Anwendungsszenarien

1. Name
2. Use Case Nummer
3. Anwendungsbereiche
4. Status
5. Ziele
6. Voraussetzungen
7. Auslösendes Ereignis
8. Beschreibung
9. Beispielhafter Einsatz
10. Ergebnis
11. Interne Hinweise
12. Akteure
13. Nebeneffekte
14. Aufwand Umsetzung
15. Finanzieller Impact

16. Quelle / Referenz
17. Ansprechpartner
18. Fachgruppen
19. Kommerzielle Produkte und Anbieter

Onlinematerial Nr. 6: Liste der Aufgabenprofile

Nr.	Aufgabenprofil	Existierende Aufgabenprofile	Existierende Fähigkeiten & Tätigkeiten	Veränderte/neuartige Fähigkeiten & Tätigkeiten
**1. Strategie**				
1.1	Chief Medical Information Officer	– Führungskräfte im Krankenhaus mit Digitalisierungs-Kenntnissen	– Klinische Sicherheit in IT-Projekten<br>– Medizinisches Fach- oder Erfahrungswissen	– Klinische Sicherheit in KI-basierten IT-Projekten<br>– Entwicklung & Umsetzung einer KI-Strategie
1.2	Expert*in für Datenintegration	– IT-Mitarbeitende	– Erfahrung im Umgang mit Daten aus medizinischen Geräten und Software<br>– Vernetzung von Systemen	– Kenntnisse zu Datenmanagementsystemen<br>– Kenntnisse zu medizinischen Datenformaten und technischen Schnittstellen<br>– Umsetzung von Anforderungen für Data-Science-Pipelines
1.3	Expert*in für KI-Recruiting und Weiterbildung	– Personalbeauftragte*r aus den Bereichen Qualifikation und Recruiting	– Identifikation und Anwerbung von neuen Mitarbeitenden<br>– Identifikation von Qualifizierungsbedarfen<br>– Entwicklung und Umsetzung von Qualifizierungs-Maßnahmen	– Recruiting mit Fokus auf digitale Skills<br>– Entwicklung und Umsetzung von Qualifizierungs-Maßnahmen mit Fokus auf den Themen digitale Medizin, medizinische Daten und Systeme
**2. Klinisches Personal**				
2.1	Einsatz von klinischer Entscheidungs-Unterstützung	– Ärztin/Arzt<br>– Pflegekraft	– Medizinisches Fachwissen<br>– Patient*innen-Wissen<br>– Klinische Erfahrungswerte	– Einsatz der elektronischen Patientenakte (ePA)<br>– Je nach Fachgebiet:<br>o Einsatz von CDSS in Diagnose und Therapie<br>o Einsatz von digitalen Medikationsassistenten<br>o Einsatz von digitalen Order-Entry-Systemen<br>o Einsatz digitaler Befundungsunterstützung

				o Verwendung von Digitalen Gesundheitsanwendungen (DiGAs)<br>– Kombination von medizinischem Wissen, Erfahrungswerten und CDSS zu Entscheidungen<br>– Erstellung einer Zweitmeinung auf Basis von Erstmeinungen von KI-Algorithmen und kritischer Datenbeurteilung<br>– Kenntnisse über Funktionsweise und Einschränkungen von CDSS<br>– Evaluation und kritisches Hinterfragen von Ergebnissen aus CDSS<br>– Vermittlung von Ergebnissen aus CDSS an Patient*innen und Einbeziehen der Patient*innen in die Entscheidungsfindung bzw. den Entscheidungsprozess („Shared Decision Making“)
2.2	Telechirurg*in, Robotik-Chirurg*in	– Chirurg*in	– Chirurgische Fähigkeiten<br>– Medizinisches Fachwissen<br>– Patient*innen-Kommunikation<br>– Klinische Erfahrungswerte	– Anwenderkenntnisse im Bereich Robotik<br>– Arbeit mit AR-Technologien und 3D-Technologien<br>– Kenntnisse zu Remote Surgery<br>– Einsatz der elektronischen Patientenakte (ePA)
2.3	Telemedizinische & Quantified-Self-Gesundheitsberatung	– Gesundheitsberatung (u.a. Ernährungsmediziner, Diät- bzw. Ernährungsberater, Health Coach, Fitnesstrainer, Schlafmediziner)	– Medizinisches Fachwissen (z.B. über Physiologie, Anatomie, Ernährung, Fitness)<br>– Managementfähigkeiten<br>– Selbstorganisation<br>– Soziale Kompetenzen, Empathie	– IKT-Kenntnisse<br>– Quantified-Self-Kenntnisse, Kenntnisse zu Mobile Health<br>– Einsatz der elektronischen Patientenakte (ePA)
2.4	Expert*in für KI-basierte Medizinprodukte	– Ärztin/Arzt<br>– Pflegekraft	– Einsatz, Kontrolle und Pflege von u.a. Prothesen, Orthesen, Messgeräten, Defibrillatoren und Pumpen<br>– Medizinische Pflege<br>– Soziale Kompetenzen, Empathie	– Einsatz, Kontrolle und Pflege von intelligenten Medizingeräten<br>– Auswertung von Daten aus Medizingeräten<br>– Einsatz der elektronischen Patientenakte (ePA)<br>– Einsatz von digitalen Medizinprodukten in der Pflege (z.B. Sensorik, Dekubitusmatte)<br>– Verwendung von Digitalen Gesundheitsanwendungen (DiGas) und Digitalen Pflegeanwendungen (DiPas)

				– Beratung und Unterstützung von Patientinnen und Patienten bei der Nutzungs von DiGas und DiPas<br>– Shared Decision Making mit Patient*innen
2.5	Telepflege, Robotikpflege	– Pflegerisches Personal	– Medizinische Pflege<br>– Soziale Kompetenzen, Empathie	– Anwenderkenntnisse im Bereich Robotik<br>– Arbeit mit AR-Technologien<br>– IKT-Kenntnisse<br>– Veränderte Interaktion mit Patientinnen und Patienten<br>– Teilnahme an digitalen Care-Konsilen<br>– Shared Decision Making mit Patient*innen<br>– Verwendung der elektronischen Patientenakte (ePA)
2.6	Expert*in für Digitale Pflegedokumentation	– Pflegerisches Personal	– Medizinische Pflege<br>– Soziale Kompetenzen, Empathie<br>– Pflegedokumentation inkl. Monitoringsysteme	– Verwendung der elektronischen Patientenakte (ePA)<br>– Kenntnisse zu digitalen System-Schnittstellen (u.a. KIS-Integration)<br>– Kenntnisse von digitalen Pflegeterminologien (LEP, ePA, etc.)<br>– Auswertung von Sensorgestützter Dokumentation<br>– Digitale Medikationssysteme
**3. Ethik**				
3.1	Datenschutz- & Datensicherheitsexpert*in	– Datenschutzbeauftragte*r<br>– Datensicherheitsbeauftragte*r	– Kenntnis und Durchsetzung von Datenschutz und Sicherheitsrichtlinien (u.a. DSGVO, BDSG)<br>– Kenntnisse zu Cybersicherheit<br>– Umgangswissen mit personenbezogenen Gesundheitsdaten	– Kenntnisse zu Datenschutz im KI-Kontext<br>– Kenntnisse zu Datensicherheit im KI-Kontext<br>– Kenntnisse zu neuen Gesetzen und Richtlinien rund um KI-Kontext
3.2	Expert*in für KI- und Datenethik	– Ethikrat	– Abwägung zwischen medizinisch-wissenschaftlichem Erkenntnisgewinn und ethischen Normen und Werten	– Kenntnisse zu Daten- & Modellbias, Automation Bias<br>– Einschätzungen zu ethischen Fragen bzgl. KI-Anwendungen
3.3	Expert*in für KI-Absicherung und -Zertifizierung	– Expert*in für Risikomanagement,	– Risikoanalyse innerhalb eines Risikomanagements	– Kenntnisse zu Autonomie, Fairness, Transparenz und Verlässlichkeit im KI-Kontext<br>– Kenntnisse zu Datensicherheit und Datenschutz

		Qualitätssicherung, Informationssicherheit	– Absicherung & Zertifizierung von IT-Systemen	
3.4	Expert*in für Patient*innen-zentrierte KI	– Expert*innen für Patient*innen-Kontakt	– Patient*innen-Kommunikation<br>– Unterstützung von ärztlicher oder pflegerischer Kommunikation	– Analyse und Design von KI mit Hinblick auf die Auswirkungen auf Patient*innen<br>– Vermittlung zwischen Expert*innen aus Medizin und Informatik<br>– Entwicklung von Anwendungsszenarien für Patient*innen-zentrierte KI
**4. Verwaltung & Controlling**				
4.1	Expert*in für KI-assistierte Codierung	– Codierfachkraft<br>– Mitarbeiter*in im Medizincontrolling	– ICD-, OPS- und DRG-Codierung von Krankenhausaufenthalten	– Prüfung von KI-basierten Codierungsvorschlägen auf Basis der Patient*innendokumentation
4.2	Expert*in für KI-assisstierte Abrechnung	– Codierfachkraft<br>– Mitarbeiter*in im Medizincontrolling	– Kommunikation mit dem medizinischen Dienst der Krankenkassen	– Prüfung von KI-basierten Vorschlägen, Priorisierungen und Prognosen
**5. Data Science**				
5.1	KI Business Analyst*in/Business Developer*in	– Business Developer*in	– Kenntnis von Geschäftsprozessen und Regularien<br>– Kommunikationstalent zur Übersetzung zwischen Technik und Geschäftswelt<br>– Unternehmensziel in Verbindung mit Datenanalysen verstehen	– Geschäftsziel & Use Cases eines Data Science-Projekts identifizieren<br>– Datenverständnis innerhalb eines Data Science-Projekts aufbauen<br>– Evaluation eines Machine-Learning-Modells in Bezug auf ein definiertes Geschäftsziel
5.2	Data Scientist	– Business Analyst, IT-Mitarbeitende	– Erstellung von Dashboards, Reports und statistischen Auswertungen	– Heterogene Daten analysieren und interpretieren nach der einer Data-Science-Methodik (z.B. CRISP-DM)<br>– Geschäftsziel & Use Cases eines Data Science-Projekts identifizieren<br>– Datenverständnis innerhalb eines Data Science-Projekts aufbauen<br>– Daten vorverarbeiten innerhalb eines Data Science-Projekts

				– Auswahl einer Modellarchitektur und Training von Machine-Learning-Modellen<br>– Evaluation eines Machine-Learning-Modells in Bezug auf ein definiertes Geschäftsziel
5.3	Machine Learning Engineer	– IT-Mitarbeitende	– Kenntnisse zu Datenintegration<br>– Kenntnisse zu Datenformaten, medizinischen Terminologien und Schnittstellen-Standards (z.B. DICOM, FHIR, SNOMED CT)<br>– Datenbank-Kenntnisse	– Datenvorverarbeitung in Data Science-Projekten<br>– Modellarchitektur-Auswahl und Training von Machine-Learning-Modellen<br>– Evaluation von Machine-Learning-Modellen in Bezug auf ein definiertes Ziel
5.4	Data Officer	– Datenbank-Admin<br>– IT-Mitarbeitende	– Kenntnisse über Datenmanagement, Datenqualität, Datenbanken<br>– Anbinden interner und externer Datenquellen<br>– Einsatz von Technologien wie MySQL, Microsoft SQL Server, Oracle, PostgreSQL oder SAP Hana<br>– Einführung und Durchsetzung von Datenstandards (FHIR, SNOMED CT etc.)	– Bereitstellung von Zugangsmöglichkeiten und Schnittstellen zu Datenquellen für die Entwicklung von KI-Anwendungen<br>– Integration von Datenquellen für Machine Learning Pipelines
5.5	Data Engineer	– IT-Mitarbeitende	– Kenntnisse über Datenmanagement, Datenqualität, Datenbanken<br>– Einführung und Durchsetzung von Datenstandards (FHIR, SNOMED CT etc.)	– Fähigkeit Datenquellen zu identifizieren und transparent zu verknüpfen<br>– Datenverständnis innerhalb eines Data Science-Projekts aufbauen<br>– Daten vorverarbeiten innerhalb eines Data Science-Projekts (ETL = Extract, Transform & Load)<br>– Datenqualität einschätzen und verbessern

				– Bereitstellung eines Machine-Learning-Modells für Personen außerhalb des Data-Science-Projekts<br>– Kenntnisse zu Data Provenance
5.6	Datenvisualisierungs-Expert*in	– Business-Analytics-Expert*in	– Verwendung von Business-Analytics-Dashboards<br>– Erstellung von Darstellungen in Berichten	– Entwicklung von Datenvisualisierungen innerhalb der Phasen „Datenverständnis“, „Datenvorverarbeitung“, „Modellauswahl“ und „Modell-Evaluation“ eines Data Science Projekts<br>– Erstellung von Datenvisualisierungen und interaktiven Dashboards für die Kommunikation von Ergebnissen an externe Stakeholder eines Data Science-Projekts<br>– Nutzung von Tools wie Tableau, D3, Shiny, ggplot und matplotlib
5.7	Machine Learning Operations Engineer	– IT-Mitarbeitende	– Deployment von Anwendungen im Krankenhaus<br>– Monitoring und Support von Software	– Deployment, Bereitstellung & Monitoring von Machine-Learning-Pipelines<br>– Kenntnisse zu Model Drift, Data Drift<br>– Kenntnisse zu Virtualisierung und APIs
5.8	Human-Computer Interaction Expert*in	– Patient*innen-Feedback	– Feedback von Patient*innen einholen<br>– User Interfaces	– Beherrschung der Mensch-Maschine-Schnittstelle<br>– Unterstützung bei Konzeption und Design von nutzerfreundlichen KI-Anwendungen<br>– Durchführung von systematischen Untersuchungen zur Verwendung von KI-Anwendungen im Krankenhaus<br>– Erfassung und Integration von Feedback
5.9	Robotik-Expert*in	– Chirurg*in	– Einsatzkenntnisse zu robotischen Systemen	– Entwicklungskenntnisse über Robotik<br>– Erfahrungen mit hybriden Operationen, digitaler OP-Planung und der Integration von Daten aus PACS-Systemen
5.10	Expert*in für Sensordaten und Internet of Things (IoT)	– IT-Fachexpert*in	– Einsatz von mobilen IT-Anwendungen und Sensorgeräten	– Interpretation und/oder Auswertung von Sensordaten<br>– Integration von mobilen IT-Geräten in die IT-Infrastruktur<br>– Applikationsentwicklung für mobile Geräte und Sensoren
5.11	Expert*in für Sprachverarbeitung	– IT-Mitarbeitende	– Kenntnisse zu medizinischer Dokumentation (z.B. Pflegedokumentation oder Arztbriefschreibung)<br>– Kenntnisse zum Thema Sprachdiktat	– Kenntnisse zu Natural Language Processing, insbesondere Text Mining und Automatic Speech Recognition<br>– Kompetenz in der Auswertung von klinischen Texten aus der elektronischen Patientenakte

5.12	Expert*in für Bildverarbeitung	– Radiolog*in	– Auswertung von Bildgebungen<br>– Erstellung von Bildbefundungen	– Kenntnisse zu Algorithmen der Bildverarbeitung, insbesondere Deep Learning<br>– Expertise für die Klassifikation, Objekterkennung, Segmentierung
5.13	Expert*in für Textverarbeitung	– Ärztin / Arzt<br>– Pflegekraft	– Erstellung von Befunden<br>– Expert*in für medizinische Dokumentation	– Kenntnisse zu Algorithmen der Textverarbeitung, insbesondere Deep Learning<br>– Expertise im Bereich der Named Entity Recognition (NER), Relation Extraction (RE) und Textklassifikation
5.14	Expert*in für KI-gestützte Ressourcenoptimierung	– Patientenmanagement<br>– Bettenmanagement<br>– Personalmanagement<br>– Apothekenmanagement<br>– Labormanagement	– Kommunikation mit Abteilungen und Fachkliniken<br>– Planung von Bestellungen, Lieferungen, Lagerplätzen	– Aufstellen von kombinatorischen Optimierungsproblemen<br>– Anwenden von Optimierungsverfahren und Approximationsverfahren, evtl. Reinforcement-Verfahren
5.15	Big Data Expert*in	– IT-Mitarbeitende	– Datenbank-Verwaltung<br>– Lastverteilung<br>– Virtualisierung	– Kenntnisse zu Big Data-Technologien (z.B. Hadoop, Spark, BigQuery, Kafka, Storm, HDFS, Hive)<br>– Kenntnisse zu SQL- und NoSQL-Datenbanken (z.B. HBase, Cassandra, MongoDB)<br>– Linux-Kenntnisse

Glossar: CDSS = Clinical Decision Support System bzw. Entscheidungsunterstützungssystem
